# Supplementary material for: Light-controlled switching of the spin state of iron(III)
Source: Nat Commun. 2018 Nov 12;9:4750. doi: 10.1038/s41467-018-07023-1 (PMC6232099; doi:10.1038/s41467-018-07023-1)
Supplement: Supplementary file 1 — Supplementary Information [file 41467_2018_7023_MOESM1_ESM.pdf]

Shankar, *et al.,* Light Controlled Switching of the Spin State of Iron(III)

1. Supplementary Methods

General Methods

All reactions were carried out in hot air-dried glassware with magnetic stirring under nitrogen atmosphere (when required) using commercially available reagent-grade solvents (dried when necessary, but without purification), and all evaporations were carried out under reduced pressure on Büchi rotary evaporator or Heidolph rotary evaporator below 50 °C, unless otherwise noted. Yields refer to chromatographically and spectroscopically homogeneous materials, unless otherwise stated. Most reagents were purchased from Sigma Aldrich, ABCR, Alfa Aesar or Merck and were used as received. Solvents (reagent grade) were purchased from Sigma Aldrich, ABCR and Merck. Specific experimental conditions are provided under each section below.

Mass spectrometry

The high resolution (HR) mass spectra were measured with an APEX 3 FT-ICR with a 7.05 T magnet by co. Bruker Daltonics. Electron impact (EI) and Matrix-assisted Laser Desorption/Ionization (MALDI) mass spectra were measured with a Biflex III by co. Bruker.

Chromatography Stationary Phases

For column chromatography purifications silica gel (Merck, particle size 0.040-0.063 mm) was used. R_f values were determined by thin layer chromatography on Polygram® Sil G/UV_{254} (Macherey-Nagel, 0.2 mm particle size).

IR Spectroscopy

Infrared spectra were measured on a Perkin-Elmer 1600 Series FT-IR spectrometer with an A531-G Golden-Gate-Diamond-ATR-unit. Signals were abbreviated with w, m, and s for weak, medium and strong intensities. Broad signals were additionally labeled with br.

^{1}H and ^{13}C{^{1}H} NMR Spectra

^{1}H and ^{13}C{^{1}H} NMR spectra were recorded on Bruker DRX 500 (^{1}H NMR: 500 MHz, ^{13}C NMR: 125 MHz) and Bruker AV 600 (^{1}H NMR: 600 MHz, ^{13}C NMR: 150 MHz) instruments. Chemical shifts are expressed in ppm (δ), using tetramethylsilane (TMS) as internal standard for ^{1}H and ^{13}C nuclei (δH =

0; δC = 0). Multiplicities of NMR signals are designated as s (singlet), d (doublet), t (triplet), q (quartet), quin (quintet), b (broad), and m (multiplet, for unresolved lines). ^{13}C NMR spectra were recorded with complete proton decoupling, on above-mentioned spectrometers.

NMR spectra were measured in deuterated solvents (Deutero). The degree of deuteration is given in parentheses. ^{1}H NMR spectra in reference to the following signals:

Chloroform-d_1 (99.8%): δ = 7.26 ppm (s)

Dichloromethane-d_2 (99.6%): δ = 5.32 ppm (t)

Acetone-d_6 (>99.5%): δ = 2.05 ppm (quin)

Switching Experiments in NMR

Long term ^{1}H NMR switching experiments were performed on a Bruker 500 MHz NMR spectrometer in acetone-d_6 (>99.5% d). To a 0.2 mM solution of $FeTPPClO_4$ (**1**) in acetone-d_6 (523 μL) in an NMR tube, DMSO-d_6 was added (2 μL), followed by a 120 mM acetone-d_6 solution of *trans* azopyridine (75 μL, final concentration of 15 mM). The NMR tube was sealed and shaken well before measurement. The ^{1}H NMR spectrum showed a characteristic low-spin signal at -14.72 ppm. The tube was then irradiated for 5 min with light of wavelength 365 nm using a custom made LED machine (12×400 mW). During irradiation, constant mixing was ensured via rotating the tube by a mechanical rotator with air cooling using an electric ventilator (fan). The NMR tube was then transferred to the spectrometer and the corresponding spectrum showed the characteristic high-spin signal at +66.8 ppm. The first cycle of irradiation was completed by irradiating the sealed tube with light of wavelength 435 nm (LED 12×380 mW) for another 5 min using the exact same set up as described above. The partial switching experiments were repeated for 1000 cycles. Each cycle consisted of the photoconversion of *cis* azopyridine to *trans* azopyridine via alternate irradiation using lights of two different wavelengths (365, 435 nm) and measurement after regular intervals. The LEDs were connected to an automated timer device and the on and off times were fixed at a constant values of 5 minutes each for each wavelength. The solution was stable even after 1000 continuous cycles of partial switching.

Photostationary States of the Phenyl Azopyridine 2

A 15 mM solution of the azopyridine in acetone-d_6 (598 μL) containing DMSO-d_6 (2 μL) in a sealed NMR tube was kept at 60 °C for 24 h. The ^{1}H NMR spectra of this sample confirmed 100% *trans* azopyridine. The sample was then irradiated for 2, 7 and 120 minutes with a light of wavelength 365 nm using the exact same set up as described above. ^{1}H NMR spectra of the sample irradiated for

2, 5 and 10 min showed negligible changes in the concentration of *cis* azopyridine, suggesting that the photostationary equilibrium (94.4±0.3% *cis*, 5.6±0.3% *trans*) was reached within 2 min. Similar irradiation of the sample with light of wavelength 435 nm again resulted in a photostationary equilibrium (24.6±0.3% *cis*, 75.4±0.3% *trans*) as shown in Supplementary Table 1. The PSS was not greatly affected by the presence of the porphyrin in the system.

Half-life ($t_{½}$) of *cis* Azopyridine (*cis*-2)

Cis azopyridines, in general, usually exhibit a similar half-life as the corresponding azobenzenes. The half-life ($t_{½}$) of *cis* azopyridine (*cis*-**2**) was measured using ^{1}H NMR. *Cis*-**2** at its photostationary equilibrium (94.4±0.3% *cis*, 5.6±0.3% *trans*) in a sealed NMR tube was measured for several days in a 500 MHz NMR spectrometer. The half-life ($t_{½}$) of *cis*-**2** was found to be extremely high (>164 days, Supplementary Figure 7) at room temperature in acetone-d$_6$ or acetone-d$_6$/DMSO-d$_6$, whereas the same in presence of the paramagnetic Fe(III) porphyrin was significantly lower (~18 days, Supplementary Figure 8).

Magnetic Susceptibility - Evans Measurements

The paramagnetic susceptibility of admixed spin complex FeTPP(acetone)$_2^+$, high-spin and low-spin complexes FeTPP(DMSO)$_2^+$ and FeTPP(azopy)$_2^+$ were determined via the standard Evans measurements using ^{1}H NMR spectroscopy. An NMR tube with a coaxial insert, both sealable, was used. For the admixed spin complex FeTPP(acetone)$_2^+$, the outer tube was filled with a 0.2 mM solution of porphyrin FeTPPClO$_4$ (**1**) in acetone-d$_6$ (530 μL) and the inner tube was filled with a 0.2 mM solution of diamagnetic ZnTPP (Zinc tetraphenylporphyrin) in acetone-d$_6$ (60 μL). ZnTPP, that is completely diamagnetic compensates the diamagnetic contribution of the porphyrin macrocycle to the observed magnetic susceptibility. For the high-spin complex FeTPP(DMSO)$_2^+$, the outer tube was filled with a 0.2 mM solution of porphyrin FeTPPClO$_4$ in acetone-d$_6$–DMSO-d$_6$ (530 μL, 598:2 v/v) and the inner tube was filled with a 0.2 mM solution of diamagnetic ZnTPP in acetone-d$_6$–DMSO-d$_6$ (60 μL, 598:2 v/v). For the low-spin complex FeTPP(azopy)$_2^+$, the outer tube was filled with a 0.2 mM solution of porphyrin FeTPPClO$_4$ containing 15 mM azopyridine in acetone-d$_6$–DMSO-d$_6$ (530 μL, 598:2 v/v) and the inner tube was filled with a 0.2 mM solution of diamagnetic ZnTPP containing 15 mM azopyridine in acetone-d$_6$–DMSO-d$_6$ (60 μL, 598:2 v/v). For the strapped high-spin complex **17**, the outer tube was filled with a 0.721 mM solution of strapped porhyrin **17** in dichloromethane-d$_2$ (530 μL) and the inner tube was filled with a 0.721 mM solution of diamagnetic ZnTPP in dichloromethane-d$_2$ (60 μL). For the strapped low-spin complex **18**, the outer tube was filled with a 1.63 mM solution of strapped porphyrin **18** containing 66.8 mM 1-methylimidazole in dichloromethane-d$_2$ (530 μL) and the inner tube was filled with a 1.63 mM solution of diamagnetic

ZnTPP containing 1-methylimidazole (66.8 mM) in dichloromethane-d_2 (60 μL). The concentration of the internal standard, TMS (tetramethylsilane), was maintained constant in the inner and outer tubes. ^{1}H NMR spectra were recorded in a 500 MHz spectrometer at a constant temperature of 300 K. The sample (inner and outer tubes) was allowed to equilibrate at this temperature for at least 15 minutes before measurement. The following Supplementary Equation (1) was used to calculate the paramagnetic susceptibility from the experimentally measured shift in TMS signals between the inner and outer tubes.

$$\chi_M = \frac{3\delta f M}{4\pi f m} + \chi_M^o + \frac{\chi_M^o(d_0 - d_x)}{m} - \chi_{dia}^M \qquad (1)$$

where,

χ_M = Molar paramagnetic susceptibility in (cm^{3}mol^{-1})

δf = Frequency difference between the TMS peaks of the inner and outer tube (Hz)

M = Molecular weight of the substance (gmol^{-1})

f = Frequency of the NMR instrument (Hz)

m = Mass of the substance in 1 mL of solution (g)

χ^o_M = Mass susceptibility of the solvent (cm^{3}mol^{-1})

d_0 = Density of the solvent (g cm^{-3})

d_x = Density of the solution (g cm^{-3})

χ^M_{dia} = Diamagnetic correction to the magnetic susceptibility (cm^{3}mol^{-1})

Since all the solutions used in this experiment are dilute, the density of the solvent and the solutions may be considered equal, thereby nullifying the factor (d_o – d_x). The diamagnetic correction (χ^M_{dia}) has been taken care of by using equivalent diamagnetic components in the inner tube. Since the same solvents are used in the inner and outer tubes, the solvent correction factor χ^o could also be neglected. Thus the equation for calculating the molar paramagnetic susceptibility effectively reduces to Supplementary Equation (2).

$$\chi_M = \frac{3\delta f M}{4\pi f m} \qquad (2)$$

From the known values of molar paramagnetic susceptibility, the corresponding magnetic moment (μ_{eff}) may be obtained via Supplementary Equation (3).

$$\mu_{eff} = 2.828\sqrt{\chi_M T} \qquad (3)$$

where T = Temperature (K)

For a 0.2 mM solution of the high-spin complex $FeTPP(DMSO)_2^+$,

δf = 5.78 Hz

χ_M = 0.0138 $cm^3 mol^{-1}$

μ_{eff} = 5.76 B.M.

For a 0.2 mM solution of low-spin complex $FeTPP(azopy)_2^+$,

δf = 0.78 Hz

χ_M = 0.00186 $cm^3 mol^{-1}$

μ_{eff} = 2.11 B.M.

For a 0.2 mM solution of admixed spin complex $FeTPP(acetone)_2^+$,

δf = 3.53 Hz

χ_M = 0.00843 $cm^3 mol^{-1}$

μ_{eff} = 4.50 B.M.

For a 0.721 mM solution of 5,15-strapped iron(III) porphyrin chloride **17**,

δf = 24.8 Hz

χ_M = 0.01434 $cm^3 mol^{-1}$

μ_{eff} = 5.87 B.M.

For a 1.63 mM solution of 5,15-strapped iron(III) porphyrin methylimidazole **18**,

δf = 8.79 Hz

χ_M = 0.00258 $cm^3 mol^{-1}$

μ_{eff} = 2.49 B.M.

Relaxivity Measurements

The relaxation time of acetone and water were determined via NMR spectroscopy (Bruker AC 200) in acetone-d_6 + DMSO-d_6 + 1% acetone. The longitudinal (or spin-lattice) relaxation time (T_1) of acetone and water were obtained by an inversion recovery pulse sequence. The integral of the acetone and

water signals were observed as a function of the delay time (Supplementary Figure 8). See Supplementary Table 3 for the calculated values of T_1.

The transverse (or spin-spin) relaxation time T_2 was determined by a spin echo pulse sequence. The integral of the DMSO signal was observed as a function of the spin echo (n) with an echo time (τ) of 10 ms (Supplementary Figure 12). The efficiency of paramagnetic ions in shortening the relaxation time of solvent protons may be determined based on relaxivity (R_1 and R_2). The plot of the relaxation rate ($1/T_1$ or $1/T_2$) versus the concentration of the paramagnetic species shows a linear relation and the slope is defined as relaxivity, R_1 and R_2.

UV-vis and far-Visible Spectroscopy

UV-vis and far-visible absorption spectra were recorded on a Perkin-Elmer Lambda-14 spectrophotometer using quartz cells of 1 cm path length. Spectrophotometric grade solvents (2.0 mL) were employed in optical spectroscopic measurements. Irradiation experiments were performed in acetone containing small amounts of DMSO-d_6 (25.87 mM). The temperature during every measurement was fixed at 25 °C using a water-flow system connected to a thermostat.

Photostationary States of Azopyridine 2

50 μM and 1.0 mM solution of the azopyridine in acetone/DMSO (2 mL, 25.87 mM) in sealed quartz cells were irradiated for 2 min with a light of wavelength 365 nm, with stirring. The UV-vis spectrum was recorded. Extended irradiation (up to 10 min) showed negligible changes in the spectrum of *cis* azopyridine (predominant species), suggesting that the photostationary equilibrium was reached within 2 min. Similar irradiation of the sample with a light of wavelength 435 nm again resulted in a photostationary equilibrium as shown in Supplementary Figure 18. See Supplementary Table 1 for the *cis*/*trans* ratio in the photostationary state determined by ^{1}H NMR.

Far-Visible Spectroscopy of the Spin-State Changes

To a solution of FeTPP(acetone)$_2^+$ at room temperature, increasing amounts of DMSO-d_6 was added and shaken well. The final concentration of the porphyrin was fixed at 0.1 mM and the final volume at 2.0 mL. The optical spectra were recorded for each addition of DMSO-d_6, 3 minutes after placing the quartz cells in the measurement chamber for temperature equilibration. Near isosbestic point was observed around 626 nm, suggesting transformation from FeTPP(acetone)$_2^+$ to FeTPP(DMSO)$_2^+$, with intermediates having the similar optical characteristics as either of the species in solution. The process was followed by plotting the corresponding change in absorption at 686 nm at each concentration of DMSO added – the intensity increased with successive addition of DMSO at least until a final DMSO concentration of 10 mM and levelled off thereafter.

To a solution of porphyrin $FeTPP(acetone)_2^+$ at room temperature containing DMSO-d_6 (25.87 mM), increasing amounts of *trans* azopyridine were added and shaken well. The final concentration of the porphyrin was fixed at 0.1 mM and the final volume at 2.0 mL. The optical spectra were recorded for each addition of *trans* azopyridine, 3 minutes after placing the quartz cells in the measurement chamber for temperature equilibration. An isosbestic point was observed around 621 nm, suggesting a transformation from $FeTPP(DMSO)_2ClO_4$ to $FeTPP(azopy)_2ClO_4$, without any intermediates or with intermediates having the same optical characteristics as either of the species in solution. The process was followed by plotting the corresponding change in absorption at 686 nm at each concentration of *trans* azopyridine added – the intensity was found to decrease with successive addition of azopyridine at least until a final concentration of 7 mM and levelled off thereafter.

Calculation of Apparent Equilibrium Constants

The optical changes accompanying the spin state changes were used to derive the corresponding apparent equilibrium constants. The model reactions as shown in Supplementary Figure 23 were assumed and validated using the excel tool for equilibrium speciation (EST, Equilibrium Speciation Tool)[1] based on Newton-Raphson method and the reported hybrid generic algorithm, in combination with Excel's Solver.

To determine the relevant species in acetone, the change in the phenyl shifts of the porphyrin, upon addition of increasing amounts of acetone-d_6 to a solution of 0.2 mM $FeTPPClO_4$ in CD_2Cl_2, was analysed, using binding models for both, 1:1 and 2:1 complexation. The pyrrole protons were not visible over the complete range of the titration, and thus could not be used for analysis (See Supplementary Figure 21 and Supplementary Table 4).

Formation of $FeTPP(acetone)_2^+$ via $FeTPP(acetone)ClO_4$ with K_1''=0.865 L mol^{-1} and K_2''=1.077 L mol^{-1} is the most likely model. The observed and calculated shifts are given in Supplementary Table 4 and are depicted in Supplementary Figure 24. The composition of the corresponding solutions is given in Supplementary Table 5 and shown in Supplementary Figure 25. Based on these results the main component in pure acetone-d_6 (13600 mM) is complex $FeTPP(acetone)_2^+$ (~93%, see supplementary Table 5).[2]

Absorption changes for the titration of a 0.1 mM solution of $FeTPPClO_4$ in acetone with DMSO-d_6 (see Supplementary Figure 19) were analysed using both, 1:1 and 2:1 binding models as well as different assumptions for the absorption of the intermediate species $FeTPP(acetone)(DMSO)^+$ in the 2:1 binding model.

Several binding models have been tested and their fitting has been compared: a) No intermediate (mixed) complex is formed during ligand exchange (very strong cooperativity of ligand exchange). b) formation of only the mixed complex (no double ligand exchange, which is very unlikely because DMSO is known to form 2:1 complexes with $FeTPPClO_4$), c) absorption of the mixed complex $FeTPP(acetone)(DMSO)^+$ is identical to the absorption of $FeTPP(acetone)_2^+$, d) absorption of the mixed complex $FeTPP(acetone)(DMSO)^+$ is identical to the absorption of $FeTPP(DMSO)_2^+$, e) the absorption of the mixed complex is in between $FeTPP(acetone)_2^+$ and $FeTPP(DMSO)_2^+$. The SSR (sum of squared residuals) values are a) and b) $1.5 \cdot 10^{-3}$, c) $6.2 \cdot 10^{-4}$, d) $8.1 \cdot 10^{-5}$, e) $2.9 \cdot 10^{-5}$. This suggests formation of an intermediate species $FeTPP(acetone)(DMSO)^+$ according to model d) or e). Model e) is in accordance with the course of the absorption shown in Supplementary Figure 19, which shows a nearly isosbestic point at 621 nm but a different behaviour above 750 nm.

In Supplementary Table 6, the composition of the species in solution, and the observed and calculated absorption at 686 nm are given. The speciation is shown in Supplementary Figure 27. Supplementary Figure 26 shows the comparison of observed and calculated absorption. Apparent binding constants are given in Supplementary Table 10.

With the binding constants obtained from the UV-Vis experiments (K'_1=5862 L mol^{-1}, K'_2=596 L mol^{-1}), we were able to fit the shifts of the phenyl and pyrrole protons of $FeTPPClO_4$. These shifts were followed upon subsequent addition of DMSO-d_6 to a solution of $FeTPPClO_4$ in acetone-d_6 with a fixed concentration of 0.2 mM porphyrin and a fixed total volume of 600 µL (see Supplementary Table 7, Supplementary Figure 22). Fitting the NMR data with different initial assumptions for the association constants gave similar results. The best fit was obtained for K'_1=5372 L mol^{-1} and K'_2=580 L mol^{-1} (Δ_{SSR}=$4 \cdot 10^{-5}$ (sum of squared residuals)), which results in a difference of 8.4% and 2.8%, respectively. This close agreement can be interpreted as an additional confirmation of the validity of our model.

For the titration of a solution of $FeTPPClO_4$ (0.1 mM) in acetone containing DMSO-d_6 (25.87 mM) with *trans* azopyridine the binding isotherm was analysed considering both, single or double coordination of azopyridine. Occurrence of an isosbestic point at 621 nm suggested either clean transformation to a single product or formation of an intermediate $FeTPP(DMSO)(azopy)^+$ with the same optical characteristics as either species in solution (see Supplementary Figure 20). The most likely model was stepwise coordination of two azopyridines, assuming that $FeTPP(DMSO)(azopy)^+$ has the same absorption as the final complex ($FeTPP(azopy)_2$). This implicates that the first coordination of azopyridine induces spin change from high-spin to low-spin.

Titration with *trans* azopyridine was also followed by ^{1}H NMR spectroscopy, but neither the pyrrole shift (slow exchange, broad signals) nor the phenyl shifts (not observable because of excess of azopyridine) could be analysed.

A refined binding model could be obtained by fitting the data of the titration with *trans* azopyridine by taking into account the apparent binding constants for DMSO-d_6 (see Supplementary Table 10). This provided the basis to calculate the concentration of all relevant species in solution for the single titration points and for the switching experiments (see above).

Using the combined model, we were able to determine the composition of the species in the solution of the NMR switching experiments. Assuming that the binding constants of *cis* azopyridine are much lower than those of DMSO-d_6 and, consequently, that *cis* azopyridine does not bind, we determined the composition of the solutions in the photostationary states after irradiation with 365 and 435 nm, respectively (see Supplementary Tables 12 and 13). Further evidence that *cis* azopyridine is a very weak ligand, or does not bind at all, is provided in Supplementary Figure 3b. With these values, the switching efficiency could be calculated as 76.3% (see Supplementary Figure 34).

Switching Experiments in Optical Spectroscopy

To a 0.1 mM solution of $FeTPPClO_4$ in acetone, containing DMSO-d_6 (25.87 mM) in a sealed quartz cell, *trans* azopyridine (trans-**2**) (75 eq., 7.5 mM) was added. The quartz cell was sealed and shaken well before acquiring the optical spectrum in the visible region (500-800 nm). The cell was then irradiated for 2 min with a light of wavelength 365 nm, under magnetic stirring and the optical spectrum was obtained. The first cycle of irradiation was completed by irradiating the sealed cell with a light of wavelength 435 nm for another 3 min. The partial switching experiments were repeated for 10 cycles. Each cycle consisted of the photoconversion of *cis* azopyridine to *trans* azopyridine via alternate irradiation using lights of two different wavelengths (365, 435 nm) and measurement after regular intervals. Supplementary Figure 35 right shows reversible changes in absorption at 686 nm as a function of number of irradiation cycles.

Electron Paramagnetic Resonance (EPR) Spectroscopy

Sample solutions of Fe^{III} porphyrin complexes were vacuum-sealed in EPR quartz-glass tubes. X-band (≈9.5 GHz) continuous wave (CW) EPR experiments on samples in acetone at 8 K were performed with a Bruker ESP 380E spectrometer equipped with an Oxford Instruments Ltd. ITC liquid He flow system and temperature controller. X-band CW EPR spectra on samples in CH_2Cl_2 were recorded at 4.8 K using a Bruker ELEXSYS E500 spectrometer equipped with an Oxford Instruments Ltd. ESR 900

liquid He flow cryostat and an ITC503 temperature controller. All spectra presented were baseline-corrected by subtraction of a background spectrum of the resonator with an empty sample tube.

X-band EPR experiments at liquid He temperatures were performed on the various Fe^{III} porphyrin complexes dissolved in acetone or CH_2Cl_2 to verify their spin states. Fig. S36 contains spectra of the following complexes of $FeTPP^+$ at a concentration of 0.2 mM with acetone as the solvent: $FeTPP(acetone)_2^+$ (**1a**), $FeTPP(DMSO)_2^+$ (**2**), $FeTPP(azopy)_2^+$ (**3**), $FeTPP(MeOPy)_2^+$ (**4**). **1a**, **2** and **3** each exhibit a peak around g = 6 of different intensity together with a smaller, sharper peak at $g \approx 2$, indicative of either high-spin S = 5/2 states ($g_\perp \approx 6$, $g_\parallel \approx 2$) or admixed S = 3/2, 5/2 states.[3] As expected, **2** shows the most intense high-spin signal, while in **3**, its intensity is much smaller (~3% of **2**). **1a** exhibits a $g \approx 6$ signal broader than the one from **2**, of intermediate intensity, and there possibly is another, small feature around g = 4.7-4.8. This appearance indicates a contribution from an intermediate S = 3/2 spin state and thus an admixed S = 3/2, 5/2 state for **1a**. **4** does not contain any S = 5/2 component, as expected, but also no other strong signals. In the spectra of **3** and **4**, there could however be weak signals around g = 3.3 and g = 3.1, respectively of very small intensity. These g values are characteristic for so-called “large g_{max}” signals,[4,5] originating from low-spin S = 1/2 states, as expected for these complexes, of $(d_{xy})^2(d_{xz},d_{yz})^3$ electronic ground state configuration.

In pulse mode, in contrast, no signals from the $FeTPPClO_4$ complexes in acetone could be observed. The reason for this is thought to be the bad glassing properties of acetone, possibly promoting agglomeration of the complexes. Magnetic interactions between the iron centers and concomitant enhanced relaxation rates prevent that electron spin echoes can be detected, while their CW signals can still be measured.

Hence, dichloromethane (CH_2Cl_2), which possesses more favorable glassing properties, was chosen as solvent for further experiments. Fig. S37 shows X-band CW EPR spectra of complexes **3** and **4** (0.2 mM in CH_2Cl_2). As in the samples containing acetone, **3** exhibits a high-spin signal at g = 5.99, while **4** does not. Both samples show large g_{max} signals of a low-spin S = 1/2 state around g = 3.4. In Fig. S38, the EPR spectra of **3** and **4** in samples with acetone or CH_2Cl_2 as the solvent are compared. Samples with CH_2Cl_2 as the solvent did show also EPR signals in pulse mode (not shown).

Illumination of **3** with UV light of 365 nm wavelength at room temperature results in a ~20-fold increase of the component at g = 5.99 (Fig. S38), consistent with an increase of the fraction of S = 5/2 complexes by low-spin to high-spin conversion. However, there is no concomitant decrease of the large g_{max} low-spin signal, which would be expected as a result of such a process. Hence, there is

a significant overall increase of EPR signal intensity. Further illumination at room temperature with blue light of 435 nm wavelength of this sample exposed to UV light before did not lead to a decrease of the g = 5.99 signal, thus not indicating a back-conversion process from high-spin to low-spin that has been observed before for this type of sample. These results from the photoswitching experiments are not quite consistent with the light-induced behavior observed in the corresponding NMR, Evans and UV/vis experiments at room temperature. Especially, the increase of overall signal intensity upon UV irradiation suggests that not all the Fe^{III} ions present in the sample contribute to the measured EPR spectra. The reason is thought to be non-ideal complex solvation in the frozen samples, possibly resulting in cluster formation, even when using CH_2Cl_2 as the solvent.

Electrochemical Measurements

The electrochemical measurements were performed on an Autolab PGSTAT204 potentiostat equipped with a 3-electrode set-up. A platinum disk (5 mm diameter) was used as the working electrode, a platinum wire was used as the counter electrode and a Ag/$AgNO_3$ 0.01 M in acetonitrile was used as the reference electrode. The reference electrode was separated from the main cell chamber with a frit on a side-arm filled with supporting electrolyte in acetone. The cyclic voltammograms (CVs) were obtained in acetone at a porphyrin concentration of 0.2 mM. Tetrabutylammonium perchlorate (TBAP, 40 mM) was used as the supporting electrolyte. The experiments were conducted inside a glovebox at room temperature at a scan rate of 50 mV/s. All measurements were repeated at least three times to obtain reliable potential values. All the potentials are referred to the standard hydrogen electrode (SHE). The potentials were corrected using hydroxymethyl ferrocene (Fc-MeOH) as an internal reference. At the end of each measurement, 0.2 mg of Fc-MeOH was added to the solution and the midpotential was determined. The reported value of 420 mV vs SHE was used for the conversion. Upon irradiation of the solution of Fe(III)porphyrin, DMSO, *trans* azopyridine and TBAP (concentrations see Supplementary Figure 41 a) the reduction peak shifts towards a more positive potential, whereas a shift to a more negative potential would be expected if the *cis* azopyridine would not interfere with the electrochemical process. We attribute this behaviour to complexation of the reduced Fe(II) species with *cis* azopyridine. Further implications arise from the presence of high concentrations of the supporting electrolyte (tetrabutylammonium perchlorate), which is required for the electrochemical experiment. The electrolyte may perturb the equilibrium between the different spin states since it has been shown to favour the admixed state of porphyrins.[6] Independent experiments towards the elucidation of the chemistry of the Fe(II) species applying NMR, magnetic measurements, EPR and UV are currently underway and shall be reported in due course.

Syntheses

Synthesis of porphyrin 1

Tetraphenylporphyrin (TPP) and FeTPPCl were synthesized as reported.[7,8] $FeTPPClO_4$ (**1**) was prepared via a modified literature method.[9] The toluene complex obtained after crystallization from toluene was dissolved in dichoromethane (purified over basic alumina) and the solvent was removed in vacuo. This procedure was repeated several times until no toluene signals were visible in ^1H NMR.

The *high-resolution mass of the* $FeTPPClO_4$ (**1**) with different ligands:

a) Acetone: 784.24952 (Calc.), 784.24891 (Found) for $C_{50}H_{40}N_4O_2Fe$

b) *4-Methoxypyridine: 886.27132* (Calc.), *886.27059 (Found) for* $C_{56}H_{42}N_6O_2Fe$

c) DMSO: 822.17801 (Calc.), 822.17609 (Found) for $C_{48}H_{38}N_4O_2FeS_2$

Synthesis of azopyridine 2, general strategy

Azopyridine **2** was synthesized in 3 steps from commercially available 3,5-di-*tert*-butylaniline **5** and 3-amino-4-chloropyridine **8** as shown in Supplementary Figure 41. Oxidation of the aniline **5** to the corresponding nitrosobenzene **6** was achieved using oxone® in a mixture of water and CH_2Cl_2. Base-mediated coupling of the nitrosobenzene **6** with the aminopyridine **8** afforded the azo-product **9**, which on dechlorination-methoxylation resulted in the azopyridine **2**.

Synthesis of nitrosobenzene 6[10]

To a solution of Oxone® (12.0 g, 39.0 mmol) in water (100 mL) was added a solution of 3,5-di-*tert*-butylaniline **5** (2.00 g, 9.74 mmol) in CH_2Cl_2 (40 mL) and the resulting mixture was stirred at room temperature for 5 h. The formation of the nitroso compound was evident by a change in color of the solution to light green. After 5 h, the phases were separated and the aqueous phase was extracted with CH_2Cl_2 (2 x 25 mL). The organic phases were combined, dried over anhydrous $MgSO_4$ and evaporated under reduced pressure. The temperature during evaporation was maintained at 30 ^oC. The crude solid thus obtained was purified by column chromatography (silica gel, 2:5 CH_2Cl_2/pentane as eluent) to afford the nitroso compound **6** as a light green solid. 14% of the corresponding nitro compound **7** was also isolated as a yellow powder (1.84 g, 8.34 mmol, 84%).^1H NMR (600 MHz, 300 K, $CDCl_3$, TMS) δ = 7.82 (t, $^4J_{4,2}$ = 1.8 Hz, 1H, *H*-4), 7.78 (d, $^4J_{2,4}$ = 1.8 Hz, 2H, *H*-2), 1.40 (s, 18H, $C(CH_3)_3$) ppm; ^{13}C NMR (150 MHz, 300 K, $CDCl_3$, TMS): δ = 167.2 (*C*-1), 152.4 (*C*-3), 129.7 (*C*-4), 115.9 (*C*-2), 35.1 ($C(CH_3)_3$), 31.3 ($C(CH_3)_3$) ppm; HRMS (ESI) 219.16231 (Calc.), 219.16191 (Found) for $C_{14}H_{21}N_1O_1$.

Synthesis of azo compound 9

To a solution of the aminopyridine **8** (1.00 g, 7.78 mmol) in pyridine (25 mL), 60% KOH in water (75 mL) was added and the resulting mixture was heated to 80 °C. A solution of the nitrosobenzene **6** (1.80 g, 8.19 mmol) in pyridine (50 mL) was added dropwise over 15 min. The reaction mixture was then heated and kept at 80 °C for 8 h with vigorous stirring. After cooling to room temperature, the phases were separated. The aqueous phase was washed with ethyl acetate (2 x 50 mL). The organic phases were combined, dried over anhydrous $MgSO_4$ and evaporated *in vacuo*. The crude product was purified by column chromatography (1:4 ethyl acetate/cyclohexane as eluent) to obtain the azo-compound **9** as an orange solid (1.38 g, 4.18 mmol, 54%); m.p.: 93 °C; ^{1}H NMR (500 MHz, 300 K, $CDCl_3$, TMS): *trans* isomer: δ = 8.77 (s, 1H, *H*-2), 8.54 (d, $^3J_{6,5}$ = 5.3 Hz, 1H, *H*-6), 7.84 (d, $^4J_{8,10}$ = 1.8 Hz, 2H, *H*-8), 7.64 (t, $^4J_{10,8}$ = 1.8 Hz, 1H, *H*-10), 7.52 (d, $^3J_{5,6}$ = 5.3 Hz, 1H, *H*-5), 1.41 (s, 18H, $C(CH_3)_3$) ppm; *cis* isomer: δ = 8.24 (d, $^3J_{6,5}$ = 5.3 Hz, 1H, *H*-6), 7.57 (s, 1H, *H*-2), 7.36 (d, $^3J_{5,6}$ = 5.3 Hz, 1H, *H*-5), 7.23 (t, $^4J_{10,8}$ = 1.8 Hz, 1H, *H*-10), 6.75 (d, $^4J_{8,10}$ = 1.8 Hz, 2H, *H*-8), 1.17 (s, 18H, $C(CH_3)_3$) ppm; ^{13}C NMR (125 MHz, $CDCl_3$, 300 K, TMS): *trans* isomer: δ = 152.7 (*C*-7), 152.1 (*C*-9), 150.7 (*C*-6), 144.8 (*C*-3), 143.3 (*C*-4), 139.4 (*C*-2), 126.7 (*C*-10), 125.4 (*C*-5), 118.0 (*C*-8), 35.1 ($C(CH_3)_3$), 31.2 ($C(CH_3)_3$) ppm; *cis* isomer: δ = 153.2 (*C*-7), 151.9 (*C*-9), 147.8 (*C*-6), 148.8 (*C*-3), 135.8 (*C*-4), 139.4 (*C*-2), 122.7 (*C*-10), 124.5 (*C*-5), 115.0 (*C*-8), 34.9 ($C(CH_3)_3$), 31.1 ($C(CH_3)_3$) ppm; HRMS (ESI): 329.16587 (Calc.), 329.16492 (Found) for ($C_{19}H_{24}N_3Cl$); FT-IR (film): $\tilde{\nu}$ = 2963 (s), 2903 (w), 2866 (w), 1602 (m), 1562 (s), 1460 (m), 1361 (m), 1247 (m), 1187 (m), 1162 (m), 1087 (m), 882 (m), 840 (s), 746 (s), 697 (s), 680 (s) cm^{-1}; UV-vis (toluene): λ_{max} = 344 nm, log ε = 4.233 L mol^{-1} cm^{-1}.

Synthesis of azopyridine 2

Metallic sodium (3.60 g) was dissolved in MeOH (100 mL) under cooling by an ice bath. The resulting solution was added slowly to a MeOH solution (20 mL) of the azo-compound **9** (1.63 g, 4.94 mmol, the azo-compound **9** was not completely soluble in MeOH). The resulting mixture was heated to 50 °C for 2 h with vigorous stirring and was then allowed to cool to room temperature. Stirring was then continued for another 8 h and the solvent was removed under reduced pressure. Crushed ice was added to the residue and was extracted with CH_2Cl_2 (3 x 50 mL). The combined organic extracts were dried over anhydrous $MgSO_4$ and the solvent was removed under reduced pressure. The crude product thus obtained was purified by column chromatography (silica gel, 2:1 ethyl acetate/cyclohexane as eluent) to give the azopyridine **2** as a dark orange solid (1.46 g, 4.49 mmol, 91%); m.p.: 100 °C; ^{1}H NMR (500 MHz, 300 K, $CDCl_3$, TMS): *trans* isomer: δ = 8.62 (s, 1H, *H*-2), 8.54 (d ≈ s, 1H, *H*-6), 7.75 (d, $^4J_{8,10}$ = 1.8 Hz, 2H, *H*-8), 7.58 (t, $^4J_{10,8}$ = 1.8 Hz, 1H, *H*-10), 7.02 (d, $^3J_{5,6}$ = 5.7 Hz, 1H, *H*-5), 4.06 (s, 3H, OCH_3), 1.39 (s, 18H, $C(CH_3)_3$) ppm; *cis* isomer: δ = 8.23 (d, $^3J_{6,5}$ = 5.7 Hz, 1H, *H*-6), 7.78 (s, 1H, *H*-2), 7.20 (t, $^4J_{10,8}$ = 1.8 Hz, 1H, *H*-10), 6.74 (s, 1H, *H*-5), 6.73 (d, $^4J_{8,10}$ = 1.8 Hz, 2H, *H*-8),

3.73 (s, 3H, OCH_3), 1.17 (s, 18H, C(CH_3)$_3$) ppm; ^{13}C NMR (125 MHz, $CDCl_3$, 300 K, TMS): *trans* isomer: δ = 161.7 (*C*-4), 153.2 (*C*-7), 152.4 (*C*-6), 152.2 (*C*-9), 139.2 (*C*-2), 138.9 (*C*-3), 126.0 (*C*-10), 117.7 (*C*-8), 108.0 (*C*-5), 56.3 (OCH_3), 35.2 (*C*(CH_3)$_3$), 31.6 (C(CH_3)$_3$) ppm; *cis* isomer: δ = 154.9 (*C*-4), 153.9 (*C*-7), 151.4 (*C*-9), 149.6 (*C*-6), 140.7 (*C*-3), 140.5 (*C*-2), 121.9 (*C*-10), 114.4 (*C*-8), 106.7 (*C*-5), 55.4 (OCH_3), 34.8 (*C*(CH_3)$_3$), 31.2 (C(CH_3)$_3$) ppm; MS (MALDI-TOF): *m/z* = 326.06 $[M-H]^+$; HRMS (ESI): 325.21541 (Calc.), 325.21520 (Found) for ($C_{20}H_{27}N_3O$); FT-IR (film): $\tilde{\nu}$ = 2953 (s), 2869 (w), 1602 (m), 1581 (s), 1480 (m), 1361 (m), 1272 (s), 1193 (s), 1022 (s), 883 (s), 805 (s), 700 (s) cm^{-1}; UV-vis (toluene): λ_{max} = 344 nm, log ε = 4.288 L mol^{-1} cm^{-1}.

Synthesis of strapped porphyrin 17, general strategy

The ether bridge **15** was synthesized in two steps from commercially available chemicals with a yield of 24%. The *meso*-phenyl dipyrromethane **14** was synthesized with a yield of 81%. The strapped iron porphyrin **17** was prepared from **14** and **15** in two steps (see Supplementary Figures 42, 43).

Synthesis of *meso*-phenyl dipyrromethane 14[11]

Pyrrole **13** (24.0 mL, 347 mmol) and benzaldehyde **12** (850 mg, 8.00 mmol) were dissolved under nitrogen atmosphere and stirred for 15 min. TFA (150 µl) was added and the mixture was stirred at room temperature for 25 min. Subsequently, 200 ml of DCM were added and the mixture was washed with a 0.1 M potassium hydroxide solution (120 mL) and twice with water (120 mL). The combined organic layers were dried over anhydrous magnesium sulfate and the solvent was removed under reduced pressure. The crude product was purified by column chromatography (dichloromethane/cyclohexane: triethylamine (1%), 1:1, R_f = 0.61). A colorless solid was obtained (1.44 g, 6.48 mmol, 81%). ^{1}H NMR (500 MHz, $CDCl_3$): δ = 7.90 (br s, 2H, N*H*), 7.37-7.18 (m, 5H, *H*-7, *H*-8, *H*-9), 6.70-6.68 (m, 2H, *H*-4), 6.17-6.15 (m, 2H, *H*-3), 5.93-5.91 (m, 2H, *H*-2), 5.48 (s, 1H, *H*-5) ppm; ^{13}C NMR (150 MHz, 300 K, $CDCl_3$): δ = 142.2 (*C*-6), 132.4 (*C*-1), 128.6 (*C*-7) 128.5 (*C*-8), 126.9 (*C*-9), 117.2 (*C*-4), 108.4 (*C*-3), 107.2 (*C*-2), 44.1 (*C*-5) ppm.

Synthesis of 1,4-bis(2-bromoethoxy)-2-butyne 11[12]

1,4-Bis(2-hydroxyethoxy)-2-butyne **10** (1.00 g, 5.75 mmol) was dissolved in dry dichloromethane (10 mL) under nitrogen atmosphere. Subsequently, tetrabromomethane (4.20 g, 12.7 mmol) was added, then triphenylphosphine (3.30 g, 12.7 mmol) was dissolved in dry dichloromethane (20 mL) and slowly added dropwise. The reaction mixture was stirred at 0 °C for 90 min and 15 h at room temperature and afterwards poured onto mixture of dichloromethane (50 mL) and distilled water (50 mL). The combined organic layers were separated, dried over anhydrous magnesium sulfate and the solvent was removed under reduced pressure. The crude product was purified by column chromatography (cyclohexane/ethylacetate, 1:1, R_f = 0.60) to yield **12** as a yellow oil (1.70 g,

5.70 mmol, 99%). ^{1}H NMR (500 MHz, 300 K, $CDCl_3$): δ = 4.27 (s, 4H, *H*-2), 3.85 (t, ^3J = 5.5 Hz, 4H, *H*-3), 3.49 (t, ^3J = 5.5 Hz, 4H, *H*-4) ppm; ^{13}C NMR (150 MHz, 300 K, $CDCl_3$): δ = 82.3 (*C*-1), 69.7 (*C*-3), 58.5 (*C*-2), 30.0 (*C*-4) ppm.

Synthesis of the bridge 15

Salicylaldehyde (223 mg, 1.92 mmol) was dissolved in acetonitrile (20 mL) under nitrogen atmosphere. Potassium carbonate (264 mg, 1.92 mmol) and 1,4-bis(2-bromoethoxy)-2-butyne **11** (375 mg, 1.25 µmol) were added and the reaction mixture was stirred for 16 h at 80 °C. The reaction solution was concentrated and was poured onto distilled water (150 mL). The aqueous phase was extracted three times with dichloromethane (50 mL). The combined organic layers were dried over anhydrous magnesium sulfate and the solvent was removed under reduced pressure. The crude product was purified by column chromatography (cyclohexane/ethylacetate, 1:1, R_f = 0.49). A colorless oil was obtained (87.0 mg, 229 µmol, 24%). ^{1}H NMR (500 MHz, 300 K, $CDCl_3$): δ = 10.53 (s, 2H, *H*-1), 7.83 (dd, ^3J = 7.6 Hz, ^4J = 1.8 Hz, 2H, *H*-7), 7.53 (td, ^3J = 8.0 Hz, ^4J = 1.8 Hz, 2H, *H*-5), 7.03 (t, ^3J = 7.6 Hz, 2H, *H*-6) 6.99 (d, ^3J = 8.4 Hz, 2H, *H*-4), 4.31 (s, 4H, *H*-10), 4.27 (t, ^3J = 4.7 Hz, 4H, *H*-8), 3.95 (t, ^3J = 4.7 Hz, 4H, *H*-9) ppm; ^{13}C NMR (150 MHz, 300 K, $CDCl_3$): δ = 189.8 (*C*-1), 161.1 (*C*-3), 135.9 (*C*-5), 128.3 (*C-7*), 125.2 (*C*-2), 121.1 (*C*-6), 112.8 (*C*-4), 82.43 (*C*-11), 68.0 (*C*-8, *C*-9), 58.9 (*C*-10) ppm; HRMS (EI): 382.14275 (Calc.). 382.14164 (Found) for $C_{22}H_{22}O_6$; FT-IR (film): $\tilde{\upsilon}$ = 2865 (w), 1682 (s), 1597 (s), 1482 (s), 1452 (s), 1395 (m), 1350 (m), 1285 (m), 1241 (m), 1189 (s), 1161 (s), 1100 (s), 1025 (s), 925 (m), 831 (m), 755 (s), 655 (m), 606 (w), 530 (w) cm^{-1}.

Synthesis of 5,15-strapped porphyrin 16

The bridge **15** (375 mg, 983 µmol) and trifluoro boretherate (13.9 mg, 98.3 µmol) were dissolved in dichloromethane (350 mL) under nitrogen atmosphere. To this solution *meso*-phenyl dipyrromethane **14** (436 mg, 1.96 mmol), dissolved in dichloromethane (50 mL), was added under stirring over a period of 1 h. After stirring for 15 h, *p*-chloranil (504 mg, 2.05 mmol) was added and stirred for 5 h at 40 °C. Then the solvent was removed under reduced pressure and the crude product was purified by column chromatography (dichloromethane, R_f = 0.54). A purple solid was obtained (80.0 mg, 102 µmol, 10%); m.p.: 372 °C; ^{1}H NMR (600 MHz, $CDCl_3$, 300 K): δ = 8.81 (s, 8H, *H*-13, *H*-14), 8.54 (dd, ^3J = 7.2 Hz, ^4J = 1.6 Hz, 2H, *H*-9), 8.36 (s, br, 2H, *H*-*o*-Ph), 8.03 (s, br, 2H, *H*-*o*-Ph'), 7.82-7.68 (m, 8H, *H*-7, *H*-*m*-Ph, *H*-*m*-Ph', *H*-*p*-Ph), 7.50 (t, ^3J = 7.5 Hz, 2H, *H*-8), 7.07 (d, ^3J = 8.2 Hz, 2H, *H*-6), 3.72-3.69 (m, 4H, *H*-4), 2.53-2.48 (m, 4H, *H*-3), 1.06 (s, 4H, *H*-2), -2.59 (s, br, 2H, N*H*) ppm; ^{13}C NMR (150 MHz, 300 K, $CDCl_3$): δ = 159.3 (*C*-5), 142.1 (*C*-17), 134.6 (*C*-*o*-Ph'), 134.4 (*C*-*o*-Ph), 133.4 (*C*-9), 131.8 (*C*-10), 130.6 (*C*-13, *C*-14), 130.0 (*C*-7), 127.6 (*C*-*p*-PH), 126.7 (*C*-*m*-Ph, *C*-*m*-Ph'), 120.1 (*C*-8), 119.9 (*C*-15), 119.7 (*C*-16), 116.4 (*C*-12), 115.5 (*C*-11), 112.0 (*C*-6), 78.7 (*C*-1), 69.8 (*C*-4), 66.9 (*C*-3),

56.5 (*C*-9) ppm; MS (MALDI, TOF): *m/z* = 785 $[M]^+$; HRMS (EI): 784.30495(Calc.). 784.30323 (Found) for $C_{52}H_{40}N_4O_4$; FT-IR (film): $\tilde{\upsilon}$ = 2924 (w), 1596 (w), 1471 (m), 1441 (m), 1348 (m), 1284 (m), 1247 (m), 1184 (m), 1112 (m), 965 (s), 796 (s), 728 (s), 698 (s), 658 (m), 579 (m), 519 (m), 408 (s) cm^{-1}.

Synthesis of 5,15-strapped iron(III)porphyrin chloride 17

The 5,15-strapped porphyrin **16** (27.0 mg, 37.2 µmol) and iron(II)chloride tetrahydrate (180 mg, 669 µmol) were dissolved in degassed acetonitrile (30 mL) under nitrogen atmosphere and refluxed for 4 h. Then the solvent was removed under reduced pressure and the crude product was dissolved in dichloromethane (40 mL), washed twice with brine (50 mL) and water (100 mL). The combined organic layers were dried over anhydrous magnesium sulfate and the solvent was removed under reduced pressure. A brown solid was obtained (30.0 mg, 34.3 µmol, 92%); m.p.: 131 °C; ^{1}H NMR (500 MHz, CD_2Cl_2, 300 K): δ = 81.62 (s, br, 4H, *H*-pyrrole), 78.00 (s, br, 4H, *H*-pyrrole) ppm; MS (MALDI, TOF): *m/z* = 839 $[M\text{-}Cl]^+$, 874 $[M]^+$; HRMS (EI): 838.22299 (Calc.), 838.22174 (Found) for $C_{52}H_{38}FeN_4O_4$; FT-IR (film): $\tilde{\upsilon}$ = 2921 (w), 1597 (m), 1442 (m), 1336 (m), 1243 (m), 1000 (m), 801 (m), 719 (m), 659 (m), 541 (m), 463 (s), 436 (w), 418 (w), 408 (s) cm^{-1}. Due to the highly diluted NMR samples, the large number of quaternary C-atoms and the paramagnetism, ^{13}C NMR spectroscopy of **17** did not provide sufficient signal intensities. Therefore, the ^{13}C NMR spectrum was not analyzable.

Synthesis of 5,15-strapped iron(III)porphyrin, 1-methyl imidazole complex 18

The 5,15-strapped iron(III)porphyrin chloride **17** (1.42 mg, 1.63 µmol) was dissolved in 400 µL dichloromethane-d_2. To this solution 1-methylimidazole (5.48 mg, 66.8 µmol) dissolved in 80 µL of dichloromethane-d_2, was added. ^{1}H NMR (500 MHz, CD_2Cl_2, 300 K): δ = -7.92 (s, 2H, *H*-pyrrole), -10.05 (s, 2H, *H*-pyrrole), -22.15 (s, 2H, *H*-pyrrole), -23.28 (s, 2H, *H*-pyrrole) ppm; HRMS (ESI): 920.27680 (Calc.), 920.27580 (Found) for $C_{56}H_{44}N_6O_4Fe$. Due to the highly diluted NMR samples, the large number of quaternary C-atoms and the paramagnetism, ^{13}C NMR spectroscopy of **18** did not provide sufficient signal intensities. Therefore, the ^{13}C NMR spectrum was not analyzable.

2. Supplementary Figures

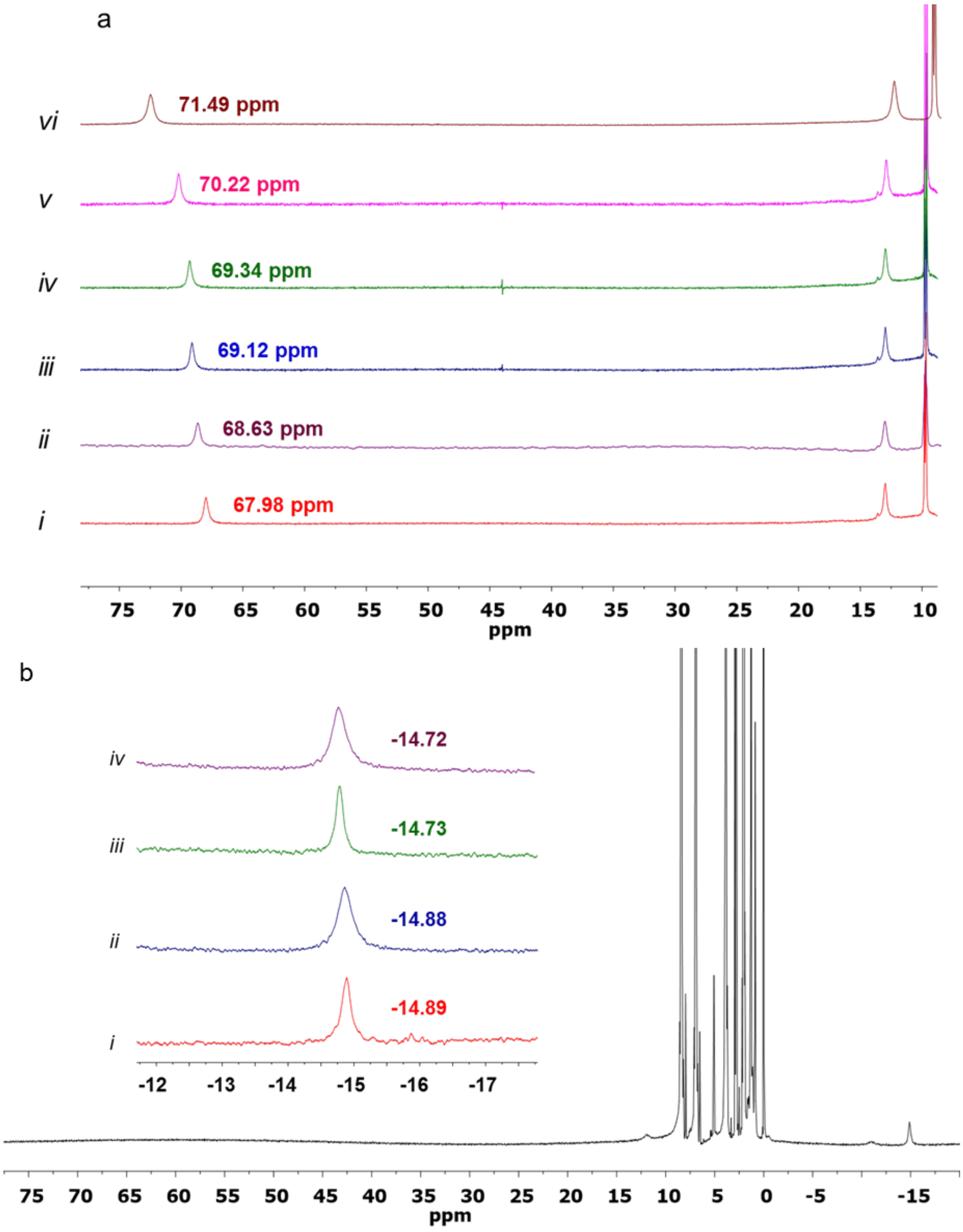

Supplementary Figure 1| Representative ^{1}H NMR spectra of the complexes obtained in this study. (**a**) The high-spin spectra obtained for the complexes formed by successive addition of DMSO-d_6 to a 0.2 mM solution of FeTPPClO$_4$ (**1**) in acetone-d_6 (600 μL total volume) at acetone/DMSO ratios of (i) 599:1, (ii) 598:2, (iii) 595:5, (iv) 590:10, (v) 500:100 and (vi) 0:600 (pure DMSO-d_6). (**b**) Typical low-spin signal obtained (i) after the addition of excess 4-methoxypyridine to a 0.2 mM solution of FeTPPClO$_4$ in acetone-d_6 (-14.89 ppm), (ii) after the addition of 70 eq. 4-methoxypyridine to a 0.2 mM solution of FeTPPClO$_4$ in acetone-d_6 containing 235 eq. DMSO-d_6 (-14.88 ppm), (iii) After the addition of excess *trans* azopyridine to a 0.2 mM solution of FeTPPClO$_4$ in acetone-d_6 (-14.73 ppm),

(ii) after the addition of 70 eq. *trans* azopyridine to a 0.2 mM solution of $FeTPPClO_4$ in acetone-d_6 containing 235 eq. DMSO-d_6 (-14.72 ppm).

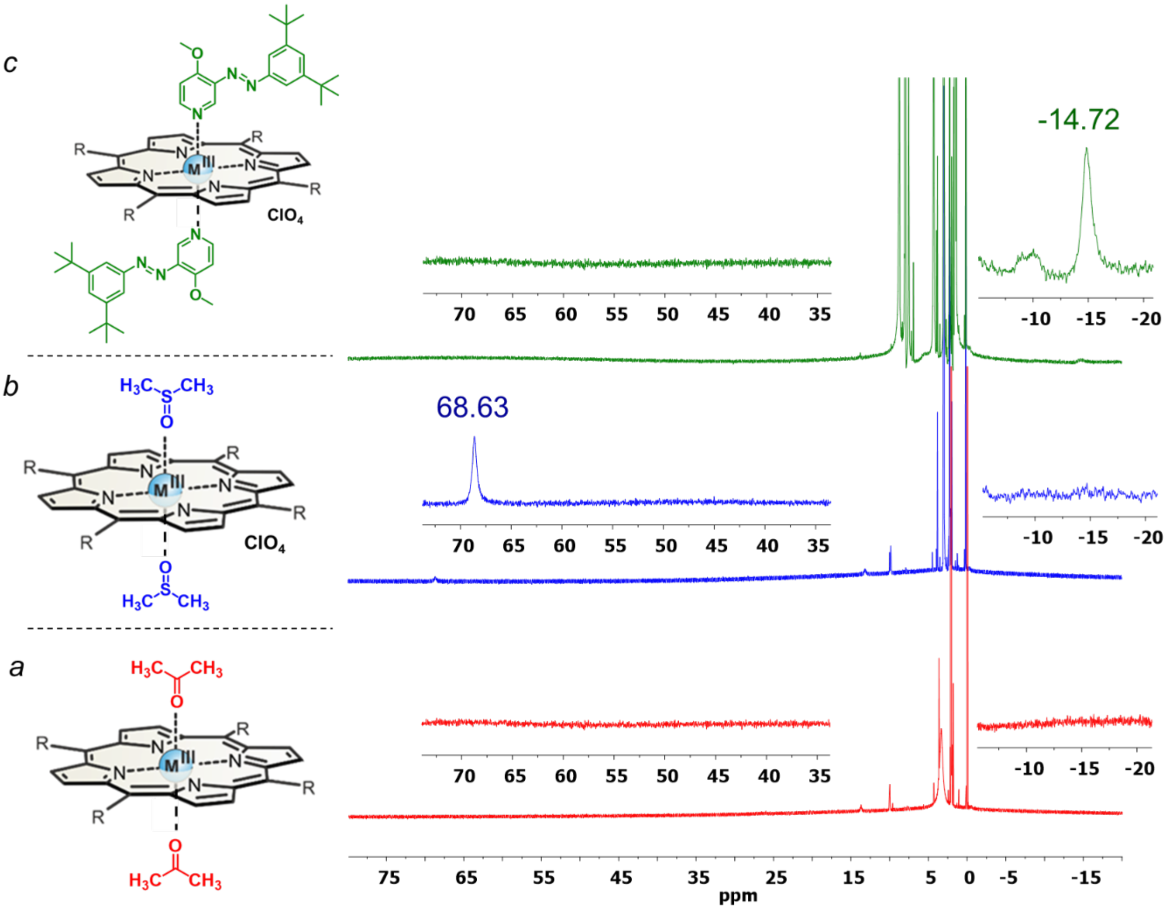

Supplementary Figure 2| ^{1}H NMR spectra showing the spin state changes involving the (a) admixed spin complex $FeTPP(acetone)_2^+$, (b) high-spin complex $FeTPP(DMSO)_2^+$ and (c) low-spin complex $FeTPP(azopy)_2^+$. NMR spectra of (a) 0.2 mM solution of the $FeTPPClO_4$ in acetone-d_6, (b) after the addition of DMSO-d_6 (2.0 μL, ~235 eq.) to a 0.2 mM solution of $FeTPPClO_4$ in acetone-d_6, and (c) after adding a 120 mM acetone-d_6 solution of *trans* azopyridine (70 eq., 75 μL, final concentration of 15 mM) to an acetone-d_6 solution (523 μL, effective concentration of 0.2 mM) of $FeTPPClO_4$ containing DMSO-d_6 (2.0 μL, ~235 eq.). The insets show the respective high-spin and low-spin regions. Similar spectra were obtained on using 4-methoxypyridine as the axial ligand at the same concentration. The spectrum of the low-spin complex obtained by adding 4-methoxypyridine or the azopyridine, *trans* azopyridine to the porphyrin solution in the absence of DMSO-d_6 also showed similar low-spin signals (-14.7 to -14.9 ppm).

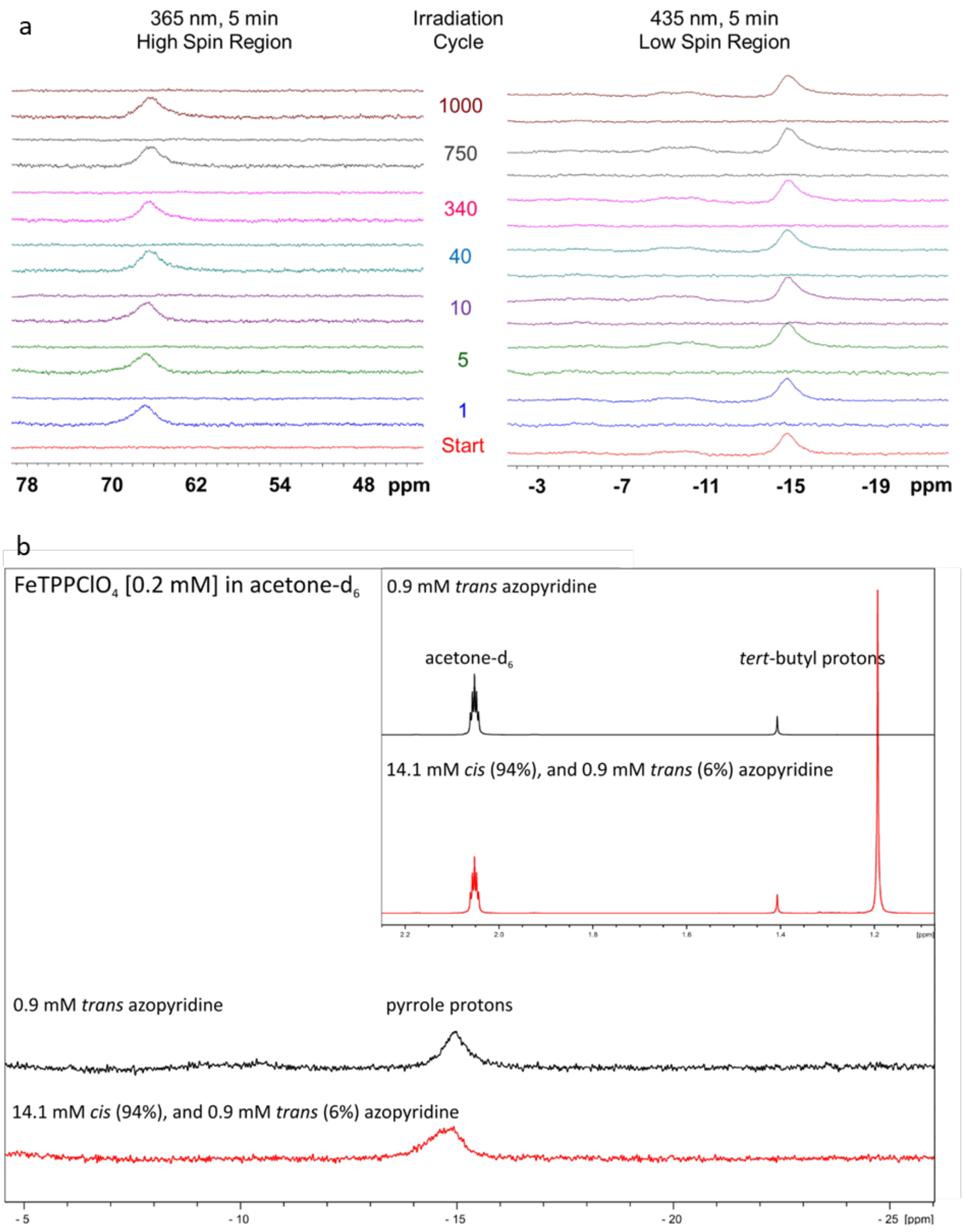

Supplementary Figure 3| (**a**) Reversible magnetic switching of $FeTPP(azopy)_2^+$. The regions of ^1H NMR spectra proving the light-induced switching of the magnetic states of $FeTPP(azopy)_2^+$ upon irradiation with lights of wavelength 365 nm and 435 nm are shown. The high-spin and low-spin states are evidenced by broad signals of the pyrrole protons at 66.8 and -14.72 ppm respectively. (**b**) Spectra of $FeTPP^+$ (0.2 mM) in acetone-d_6 (without DMSO-d_6). Black spectrum: upon addition of 0.9 mM *trans* azopyridine; red spectrum upon addition of 14.1 mM *cis* azopyridine, and 0.9 mM *trans* azopyridine (photostationary equilibrium after irradiation with 365 nm). The presence of *cis* azopyridine does not change the spectrum. Obviously cis azopyridine cannot compete even with the very weak ligand acetone-d_6 and surely is not able to replace DMSO-d_6 which is four orders of magnitude stronger than acetone as an axial ligand.

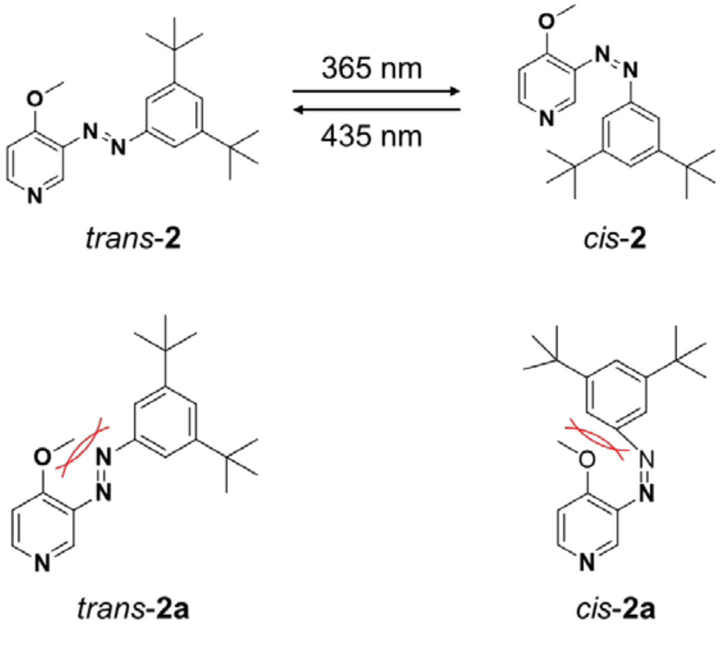

Supplementary Figure 4|Light-induced *cis-trans* isomerization of azopyridine (**2**) and the *trans* and *cis* conformers that do not form due to steric effects.

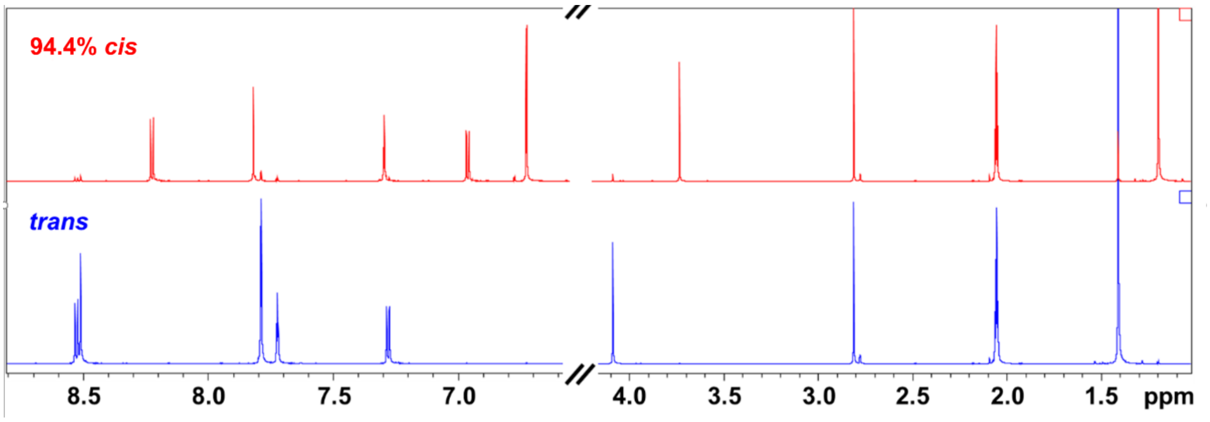

Supplementary Figure 5| ^{1}H NMR spectra of *trans* azopyridine (bottom, blue) and the photostationary equilibrium mixture of (94.4% *cis*, 5.6% *trans*) obtained after irradiating a 15 mM solution of *trans* azopyridine at 365 nm for 7 min (top, red).

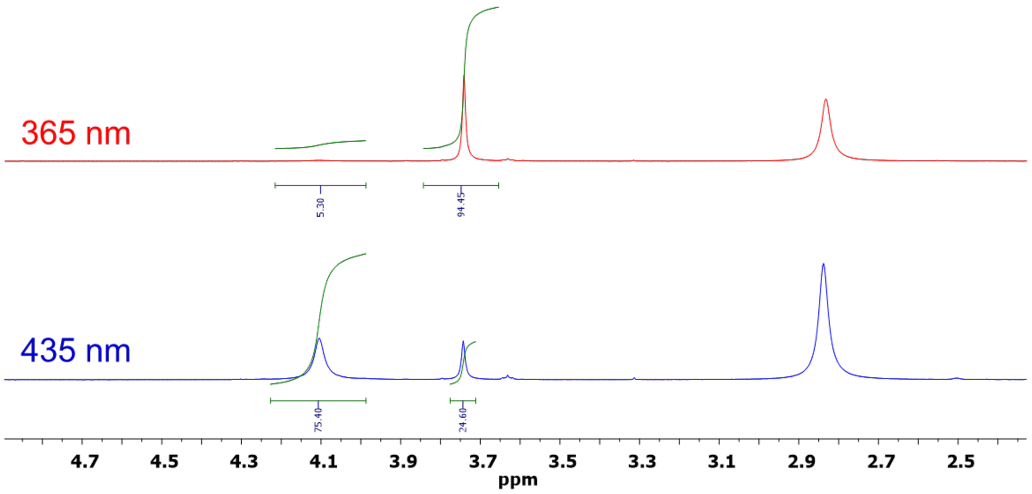

Supplementary Figure 6| ^{1}H NMR spectra of the Fe(III) porphyrin solution in acetone-d_6 (598 μL) containing DMSO-d_6 (2 μL) and the azopyridine (15 mM) irradiated at 365 nm (red, top) and 435 nm (blue, bottom). The spectra in the region δ = 2.4 – 4.9 ppm is shown. The ratio of *cis*/*trans* compound in solution is obtained by integrating the signals corresponding to –OMe protons.

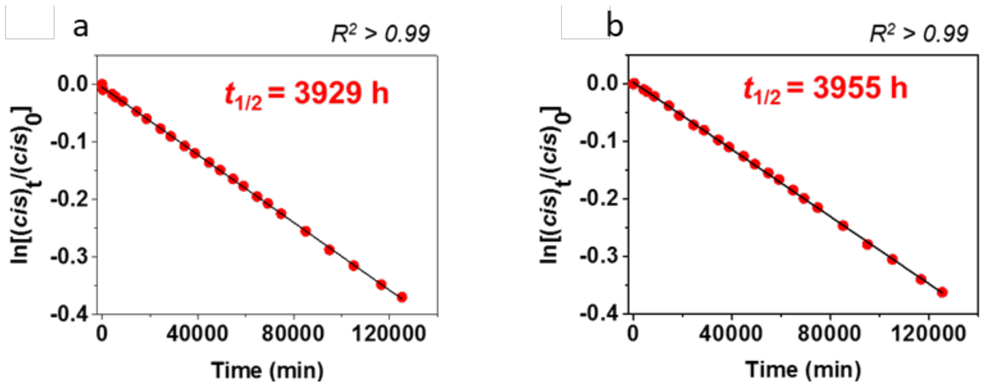

Supplementary Figure 7| Half-life ($t_{½}$) of *cis*-**2** obtained by ^{1}H NMR measurements. $t_{½}$ of 15 mM *cis*-**2** in (a) acetone (~164 days) and (b) acetone/DMSO (~164 days).

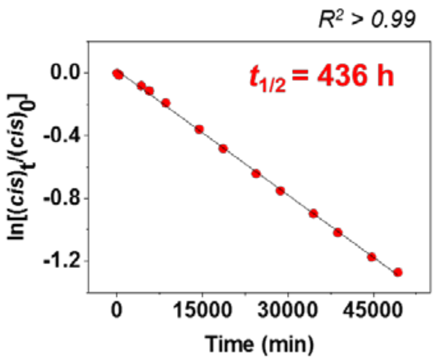

Supplementary Figure 8| Half-life ($t_{½}$) of *cis*-**2** in presence of 0.2 mM $FeTPPClO_4$ (**1**) obtained by ^{1}H NMR measurements in acetone/DMSO (~18 days).

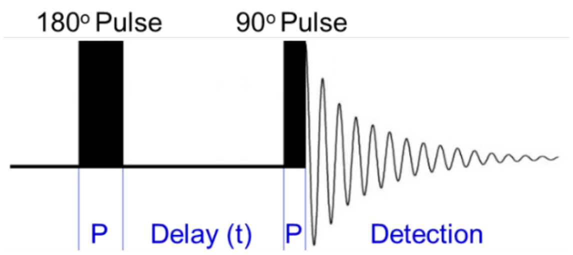

Supplementary Figure 9| Inversion recovery pulse sequence for the determination of longitudinal (or spin-lattice) relaxation time (T_1) of acetone and water.

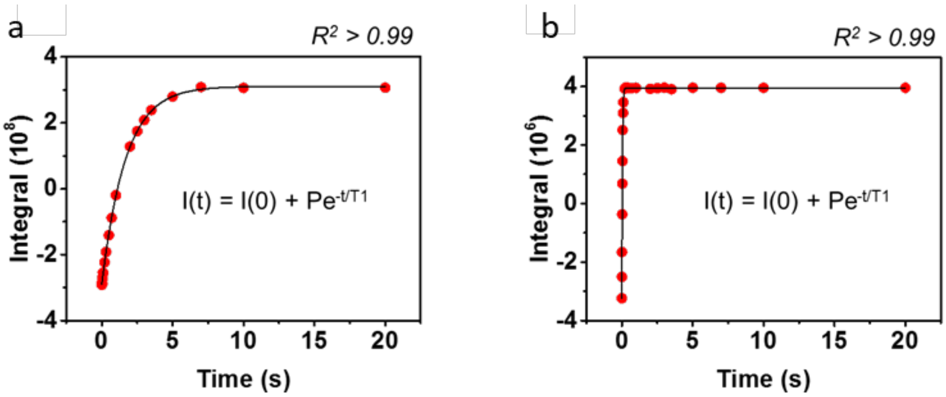

Supplementary Figure 10| The exponential fit obtained by applying the inversion recovery pulse sequence for the determination of longitudinal (or spin-lattice) relaxation time (T_1) of (a) acetone and (b) water for a 2.0 mM solution of the high-spin porphyrin $FeTPP(DMSO)_2^+$. The relaxation rates (T_1) of 1% acetone and residual water in acetone-d_6/DMSO-d_6 were measured. Similar fits were obtained for other concentrations of the porphyrin. See Supplementary Table 2 for the calculated values of T_1.

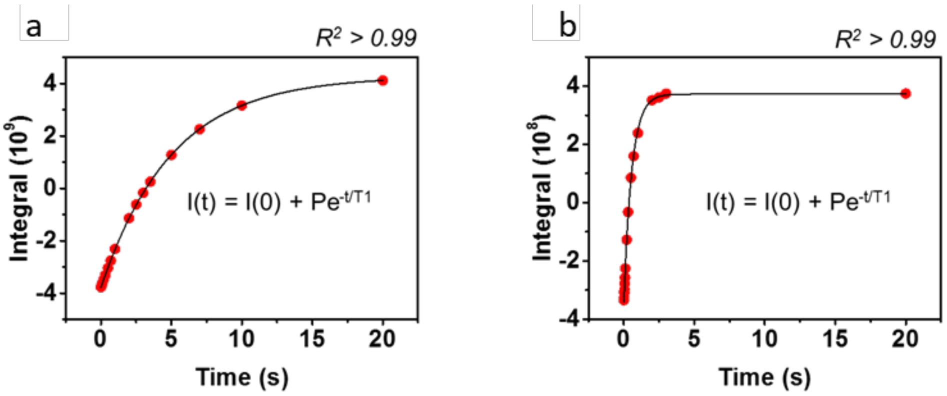

Supplementary Figure 11| The exponential fit obtained by applying the inversion recovery pulse sequence for the determination of longitudinal (or spin-lattice) relaxation time (T_1) of (a) acetone and (b) water for a 2.0 mM solution of the low-spin porphyrin $FeTPP(azopy)_2^+$. The relaxation rates (T_1) of 1% acetone and residual water in acetone-d_6/DMSO-d_6 were measured. Similar fits were

obtained for other concentrations of the porphyrin. See Supplementary Table 3 for the calculated values of T_1.

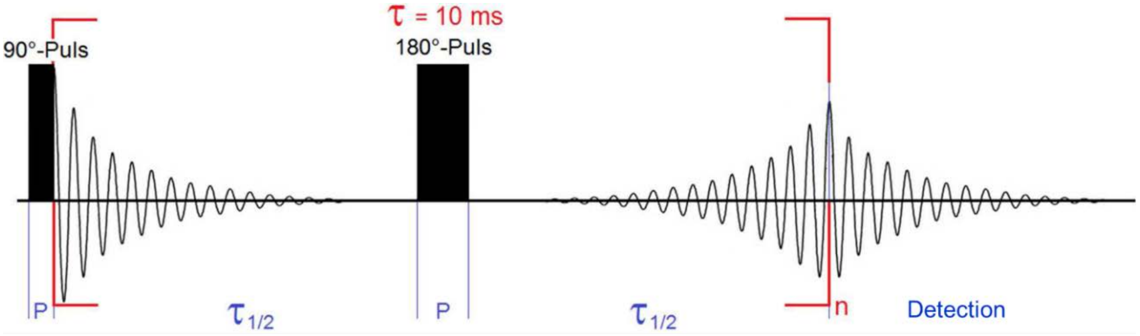

Supplementary Figure 12| Spin echo pulse sequence for the determination of transverse (or spin-spin) relaxation time (T_2) of acetone and water.

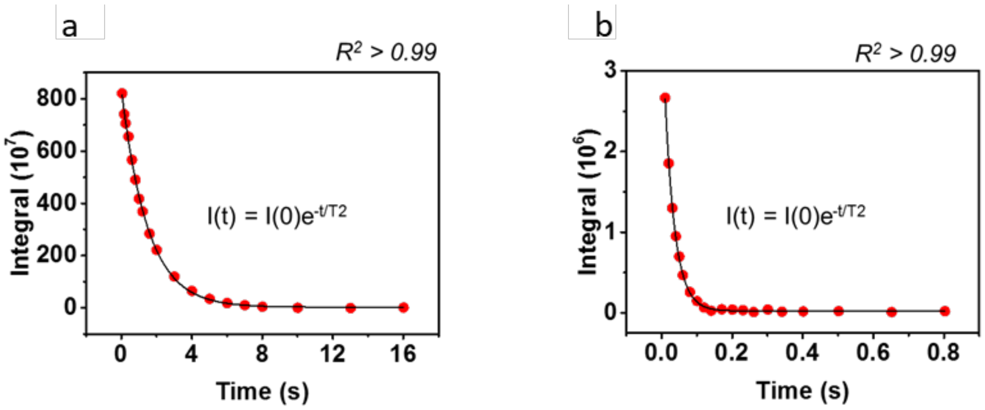

Supplementary Figure 13| The exponential fit obtained by applying the spin echo pulse sequence for the determination of transverse (or spin-spin) relaxation time (T_2) of (a) acetone and (b) water for a 2.0 mM solution of the high-spin porphyrin $FeTPP(DMSO)_2^+$. The relaxation rates (T_2) of 1% acetone and residual water in acetone-d_6/DMSO-d_6 were measured. Similar fits were obtained for other concentrations of the porphyrin. See Supplementary Table 2 for the calculated values of T_2.

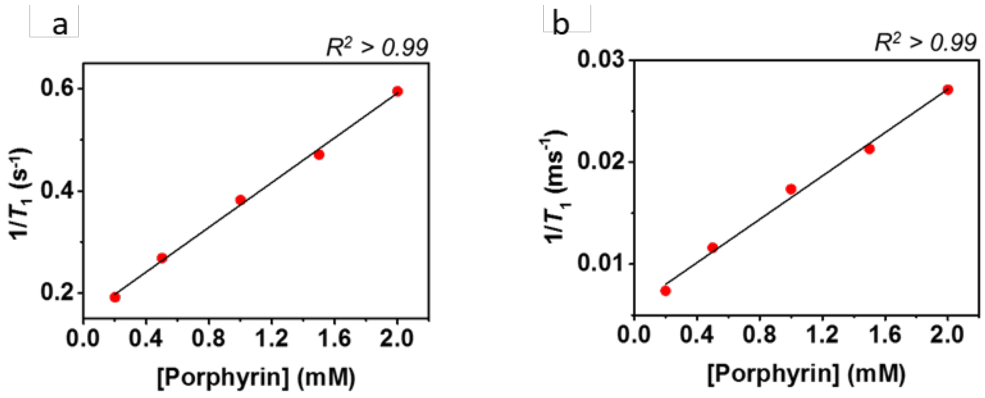

$R_1 = 0.2191 \pm 0.006\ mM^{-1}s^{-1}$

$R_1 = 0.0106 \pm 0.0004\ mM^{-1}ms^{-1}$

Supplementary Figure 14| Relaxivity plots (R_1) of (a) acetone and (b) water for the high-spin complex $FeTPP(DMSO)_2^+$. The relaxation rates (T_1) of 1% acetone and residual water in acetone-d_6/DMSO-d_6 were measured.

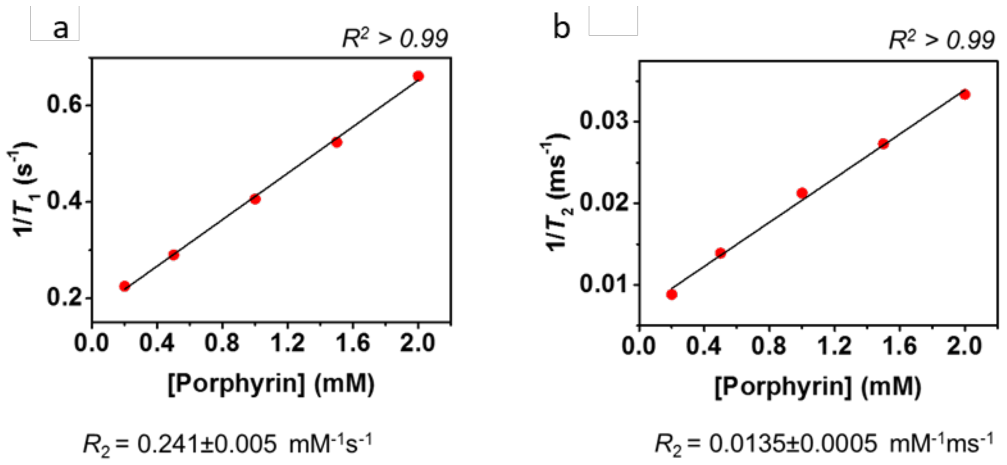

Supplementary Figure 15| Relaxivity plots (R_2) of (a) acetone and (b) water for the high-spin complex $FeTPP(DMSO)_2^+$. The relaxation rates (T_2) of 1% acetone and residual water in acetone-d_6/DMSO-d_6 were measured.

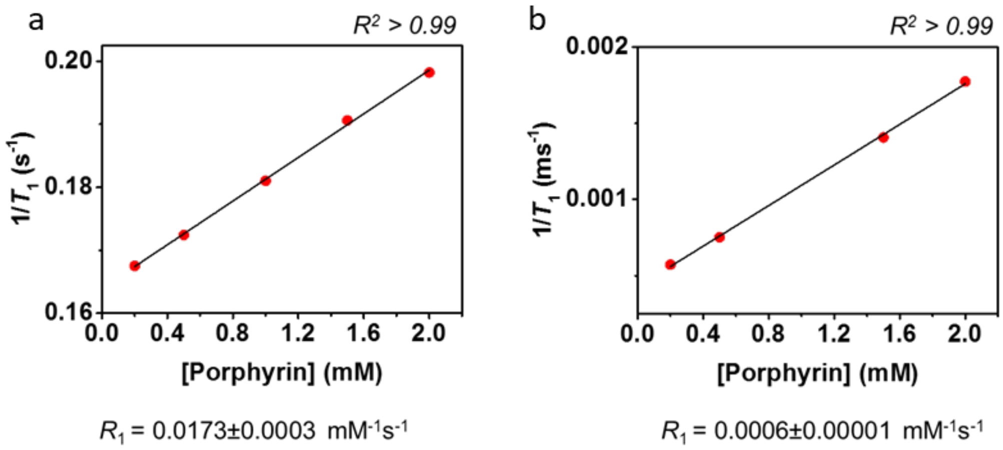

Supplementary Figure 16| Relaxivity plots (R_1) of (a) acetone and (b) water for the low-spin complex $FeTPP(azopy)_2^+$. The relaxation rates (T_1) of 1% acetone and residual water in acetone-d_6/DMSO-d_6 were measured.

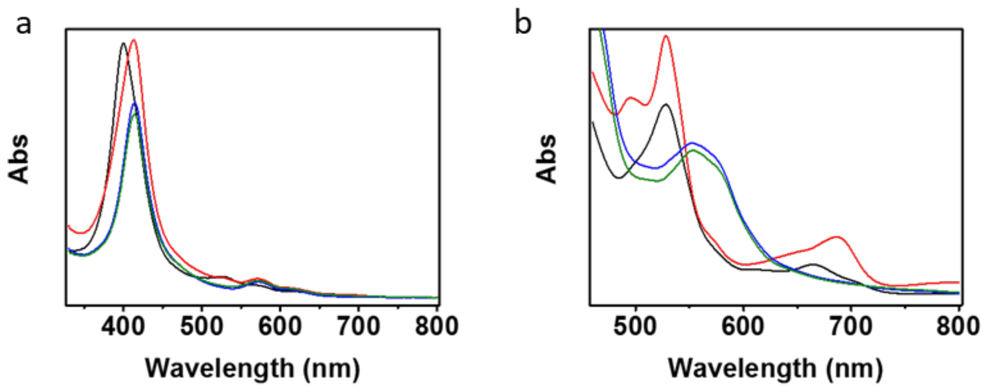

Supplementary Figure 17| UV-Vis spectra of the intermediate, high and low-spin porphyrins at concentrations of (a) 10 μM and (b) 100 μM. Color Legends: black: admixed spin complex $FeTPPClO_4$ (**1**) in acetone, red: high-spin complex $FeTPP(DMSO)_2^+$ obtained by adding ~259 eq. DMSO-d_6 to the complex $FeTPPClO_4$ in acetone ($FeTPP(acetone)_2^+$), blue: low-spin complex obtained by adding ~70 eq. 4-methoxypyridine to the complex $FeTPP(DMSO)_2^+$ in acetone containing ~259 eq. DMSO-d_6, green: pure low-spin complex obtained by adding ~70 eq. 4-methoxypyridine to $FeTPP(acetone)_2^+$ (without any DMSO-d_6). The spectra were recorded 3 minutes after placing the quartz cells in the measurement chamber for temperature equilibration. The changes observed for the spectra (blue and green) obtained by adding 4-methoxypyridine (4-OMePy) to $FeTPP(acetone)_2^+$ in the presence and absence of DMSO are nearly identical, suggesting the existence of similar species FeTPP(4-OMePy)$_2$ClO$_4$ in solution. Addition of excess DMSO-d_6 to the solution of the low-spin complex did not result in a reversible process suggesting that under these particular experimental conditions, DMSO cannot replace the coordinated 4-methoxypyridine.

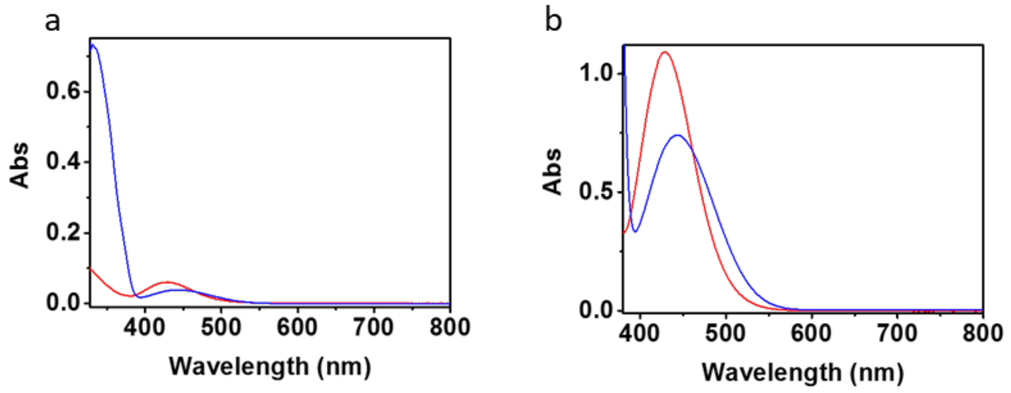

Supplementary Figure 18| UV-Vis spectra of (a) 50 μM and (b) 1 mM solution of azopyridine (acetone/DMSO) at the photostationary equilibrium obtained after irradiation using lights of wavelength 365 nm for 2 min (red) and 435 nm for 3 min (blue).

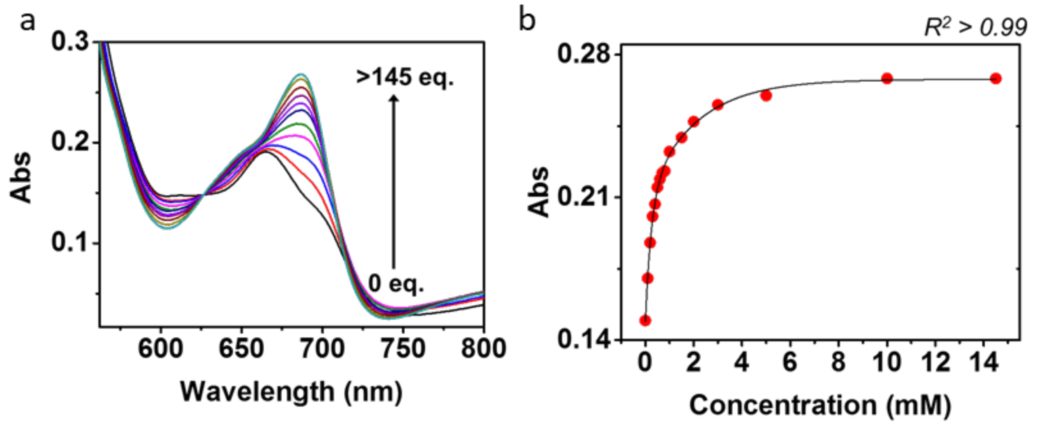

Supplementary Figure 19| (a) Change in the optical characteristics of the porphyrin upon addition of increasing amounts of DMSO-d_6 to a 0.1 mM $FeTPPClO_4$ solution in acetone.

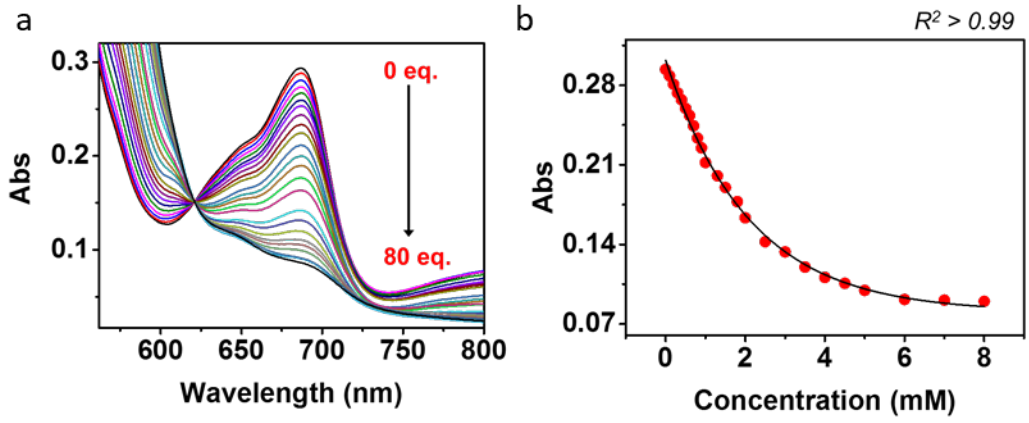

Supplementary Figure 20| (a) Change in the optical characteristics of the porphyrin upon addition of increasing amounts of *trans* azopyridine to a 0.1 *m*M $FeTPPClO_4$ solution in acetone/DMSO-d_6 (2.0 mL, 25.87 mM DMSO-d_6).

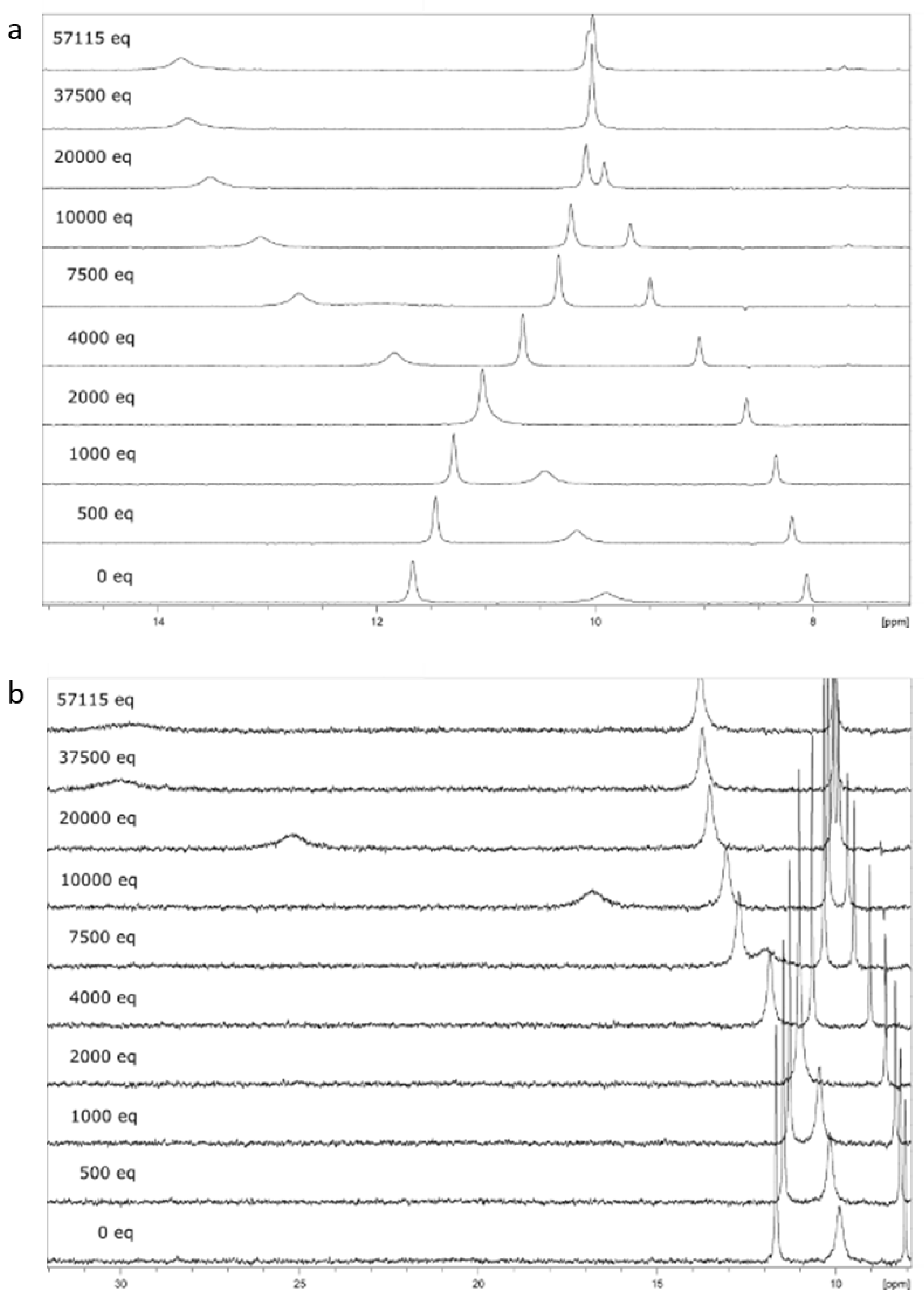

Supplementary Figure 21| (a) Change in the phenyl shifts of the porphyrin upon addition of increasing amounts of acetone-d_6 to a 0.2 mM $FeTPPClO_4$ solution in CD_2Cl_2. (b) The corresponding change in the pyrrole shifts.

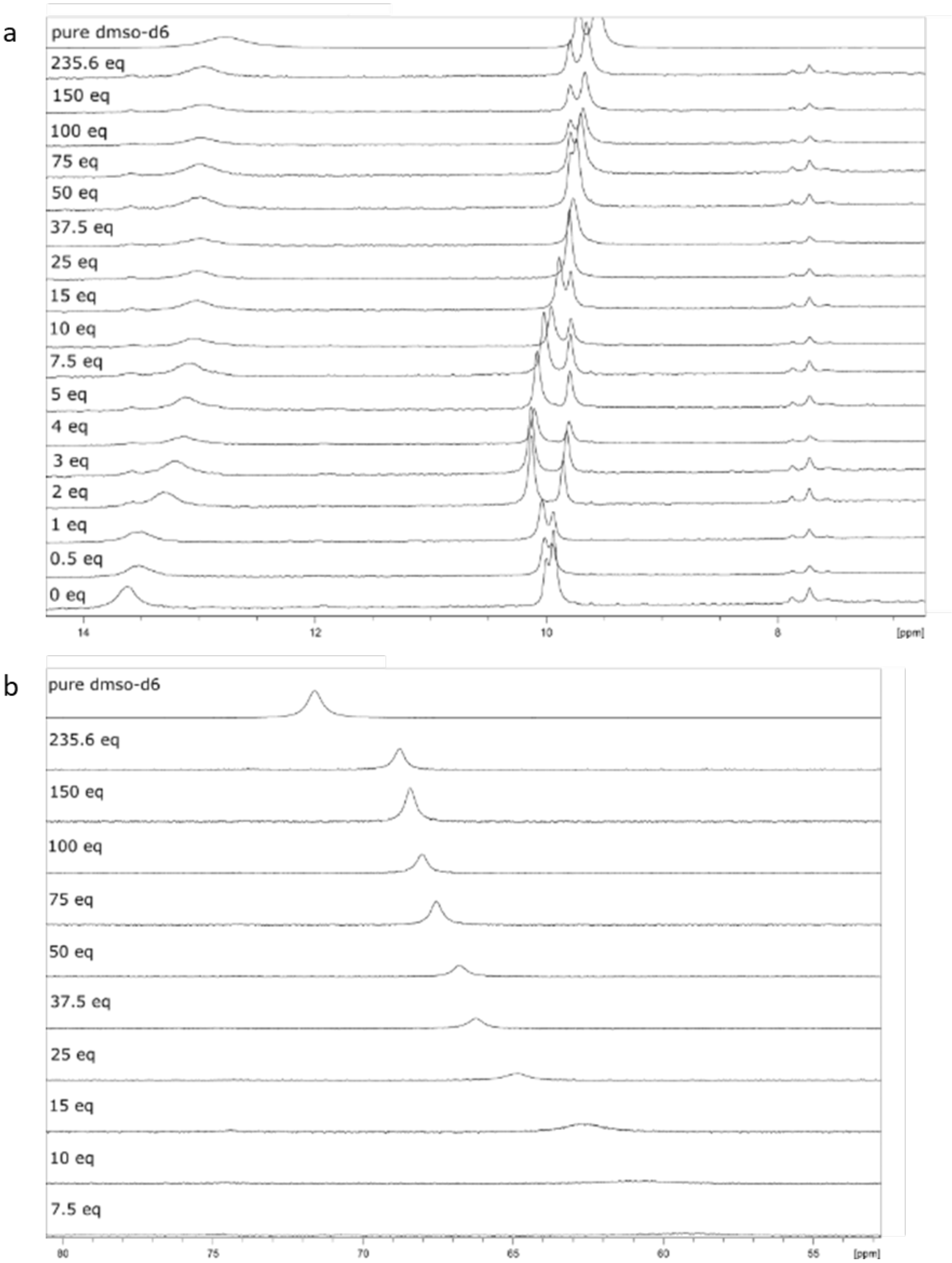

Supplementary Figure 22| (a) Change in the phenyl shifts of the porphyrin upon addition of increasing amounts of DMSO-d_6 to a 0.2 mM $FeTPPClO_4$ solution in aceton-d_6. (b) The corresponding change in the pyrrole shifts.

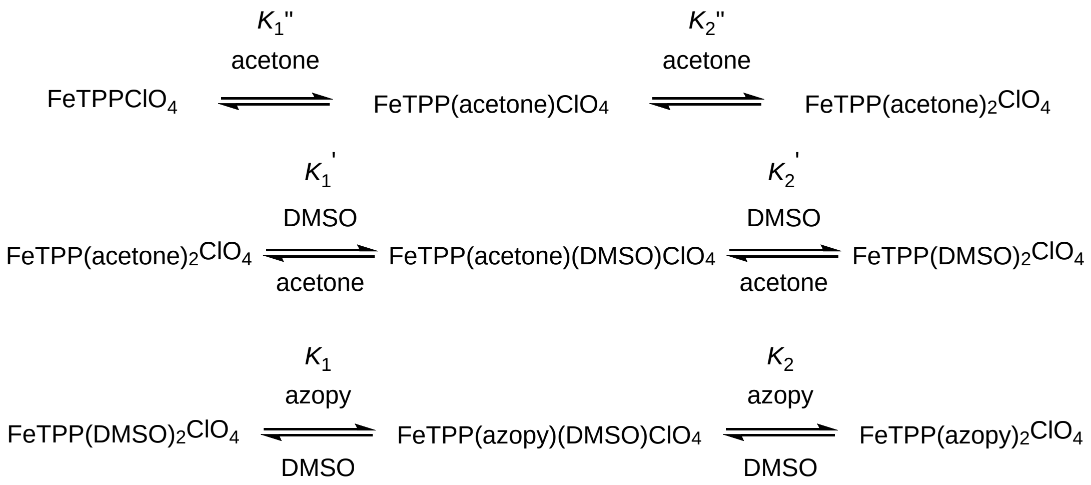

Supplementary Figure 23| Proposed scheme for the formation of the high-spin and low-spin complexes.

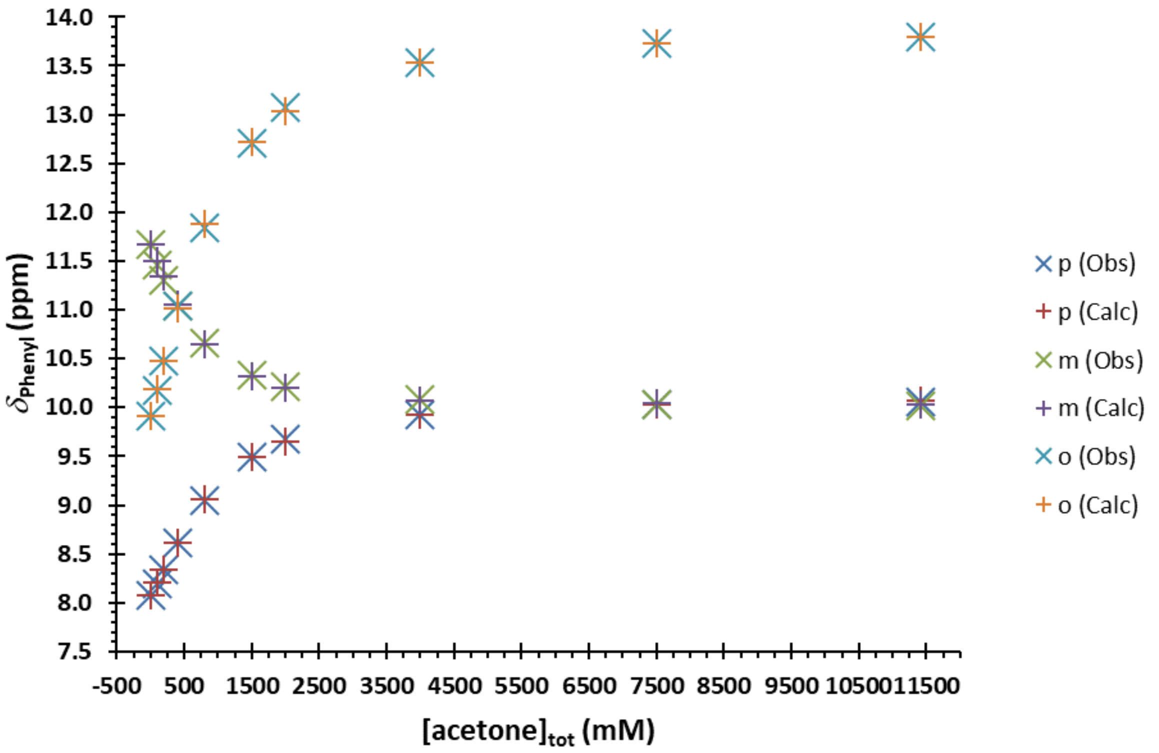

Supplementary Figure 24| Observed and calculated phenyl shifts upon titration of 0.2 mM $FeTPPClO_4$ in CD_2Cl_2 with acetone-d_6.

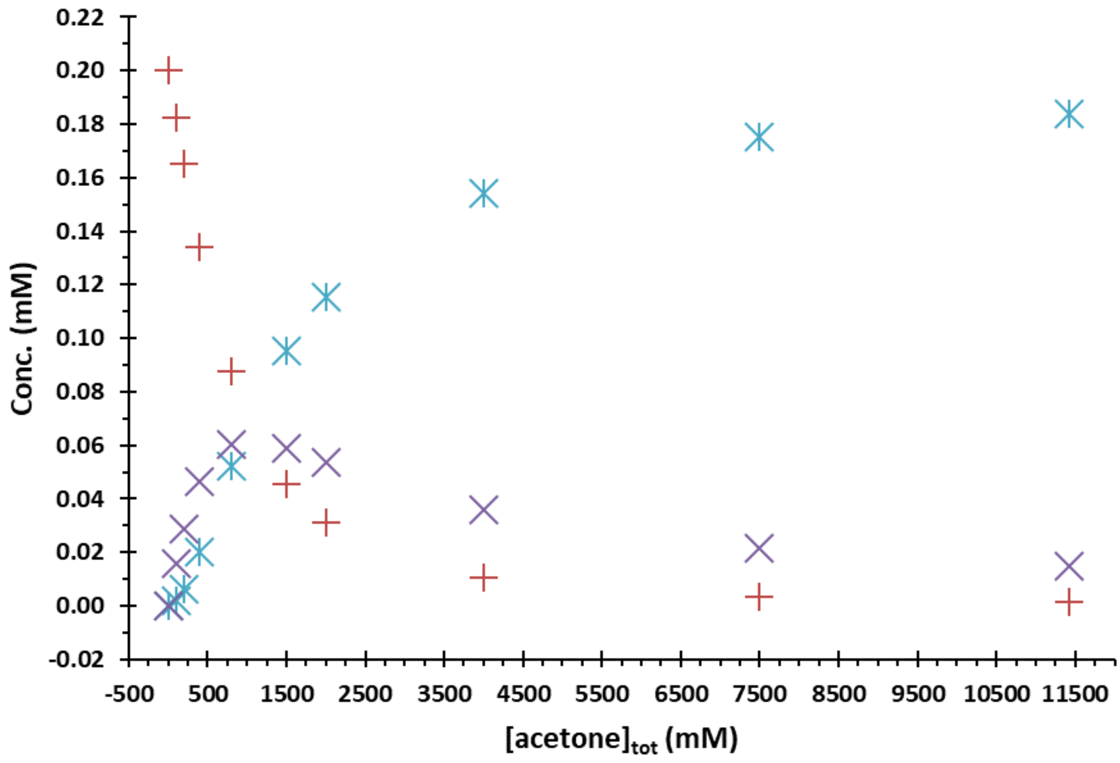

Supplementary Figure 25| Speciation plot for the titration of 0.2 mM $FeTPPClO_4$ in CD_2Cl_2 with acetone-d_6. Red: $FeTPPClO_4$, purple: FeTPP(acetone)ClO_4, blue: $FeTPP(acetone)_2ClO_4$.

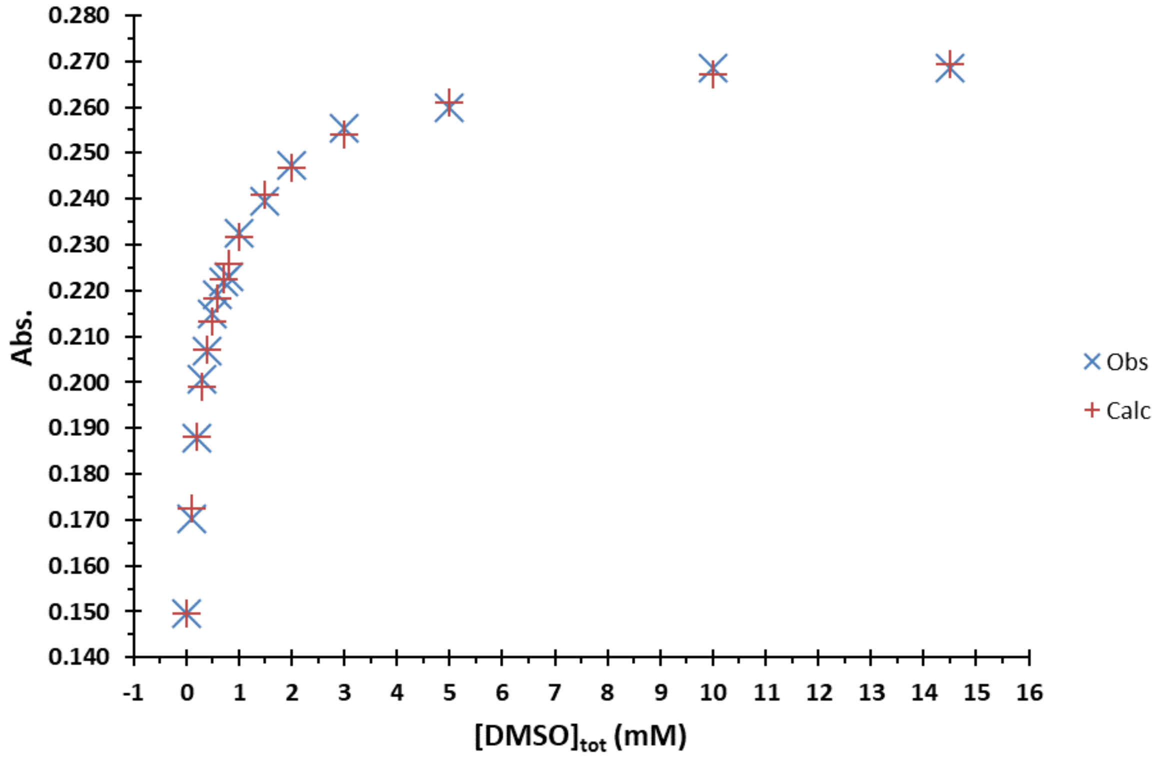

Supplementary Figure 26| Observed and calculated absorption of the solution of 0.1 mM $FeTPPClO_4$ in acetone upon titration with DMSO-d_6.

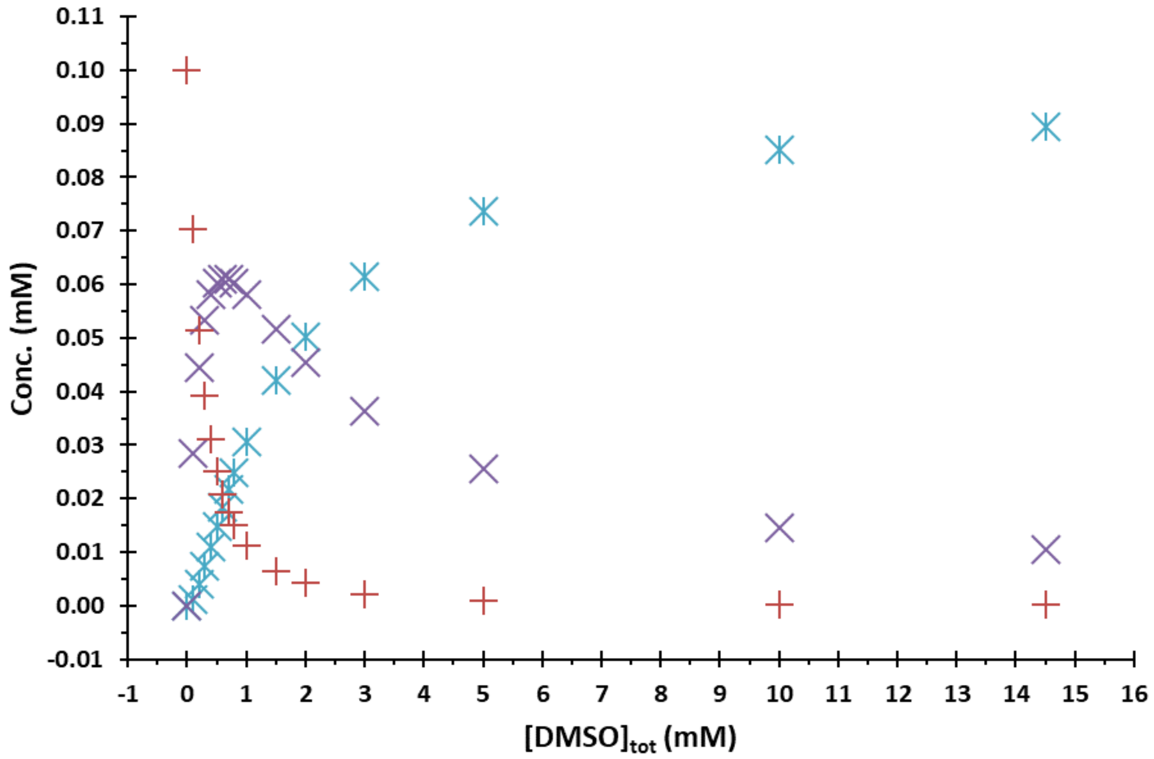

Supplementary Figure 27| Composition of the solution of 0.1 mM $FeTPPClO_4$ in acetone upon titration with DMSO-d_6. Red: $FeTPP(acetone)_2^+$, purple: $FeTPP(acetone)(DMSO)^+$, blue: $FeTPP(DMSO)_2^+$.

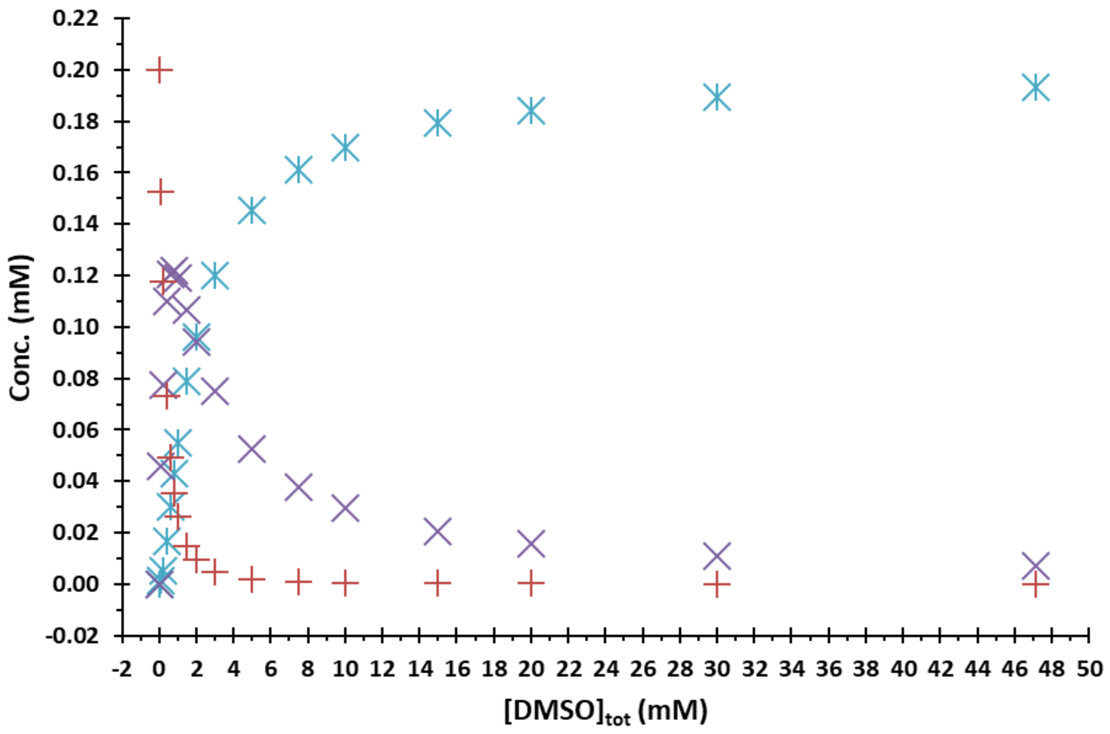

Supplementary Figure 28| Speciation plot for the titration of 0.2 mM $FeTPPClO_4$ in acetone-d_6 with increasing amounts of DMSO-d_6, utilising the binding constants derived from the UV-Vis/far-Vis titration experiments. Red: $FeTPP(acetone)_2^+$, purple: $FeTPP(acetone)(DMSO)^+$, blue: $FeTPP(DMSO)_2^+$.

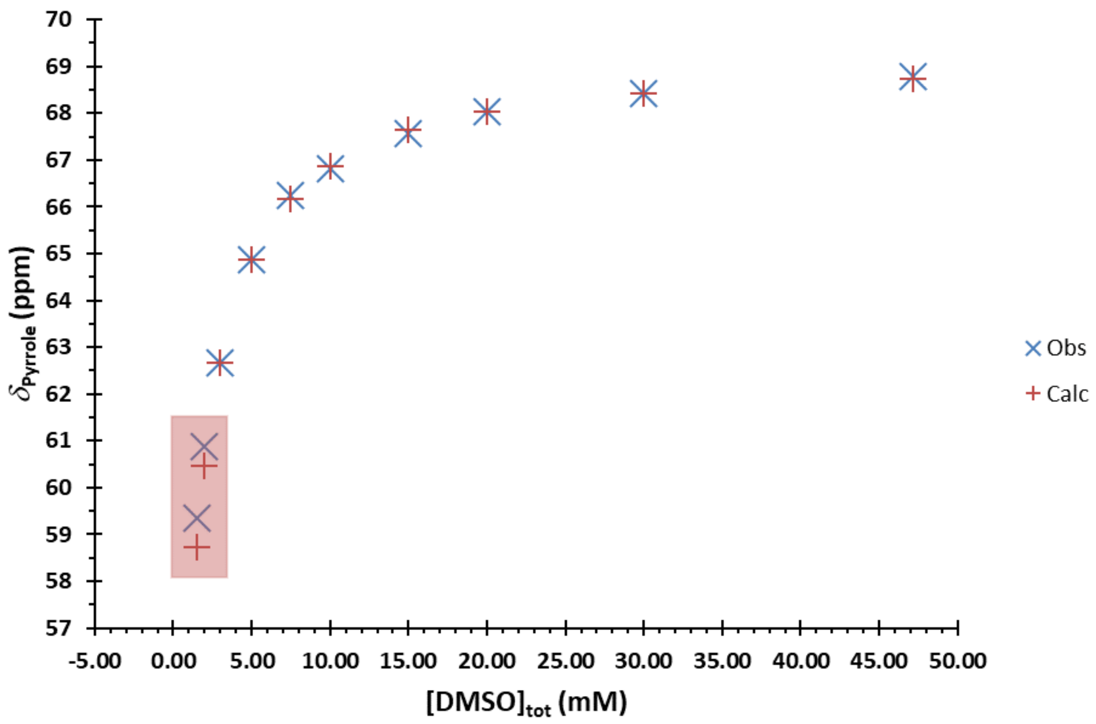

Supplementary Figure 29| Observed and calculated pyrrole shifts for the titration of 0.2 mM $FeTPPClO_4$ in acetone-d_6 with increasing amounts of DMSO-d_6, utilising the binding constants derived from the UV-Vis/far-Vis titration experiments and 40.16 ppm for the pyrrole shift in pure acetone-d_6 (See Supplementary Table 4). Red shading: observed values could not be unambiguously determined, because of severe line broadening.

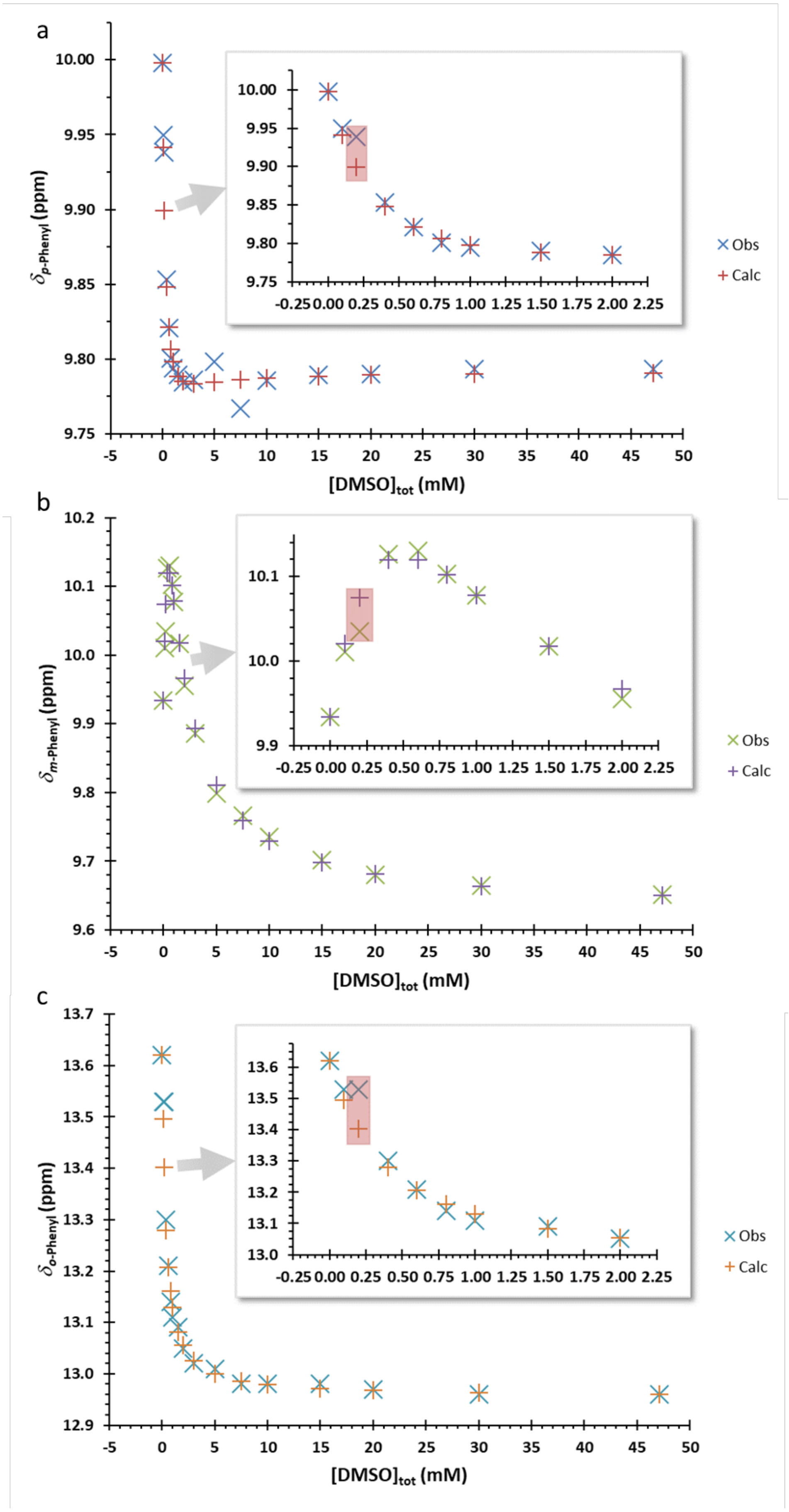
a
δp-Phenyl (ppm)
[DMSO]tot (mM)
Obs
Calc
b
δm-Phenyl (ppm)
[DMSO]tot (mM)
Obs
Calc
c
δo-Phenyl (ppm)
[DMSO]tot (mM)
Obs
Calc

Supplementary Figure 30| Observed and calculated phenyl shifts upon addition of increasing amounts of DMSO-d_6 to a solution of 0.2 mM $FeTPPClO_4$ in acetone-d_6, a) *para*, b) *meta*, (the two outliers are due to signal overlap) c) *ortho*. The inlays show the respective first nine titration points. Red rectangles show an obviously erroneous data point which was not considered for the calculation of the shifts. The binding constants derived from the UV-Vis/far-Vis experiments were used for the calculation.

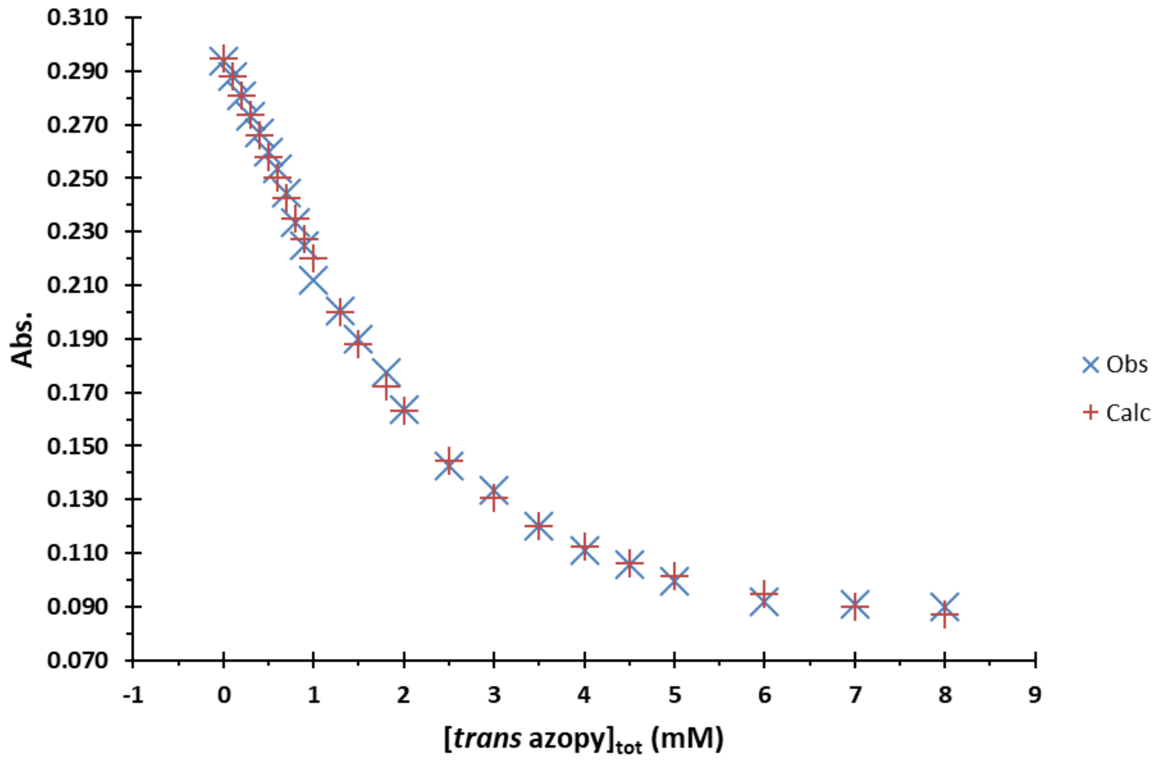

Supplementary Figure 31| Change in the absorption at 686 nm upon titration of a solution containing 0.1 mM $FeTPPClO_4$ and 25.87 mM DMSO-d_6 in acetone with *trans* azopyridine, including the binding constants of DMSO-d_6.

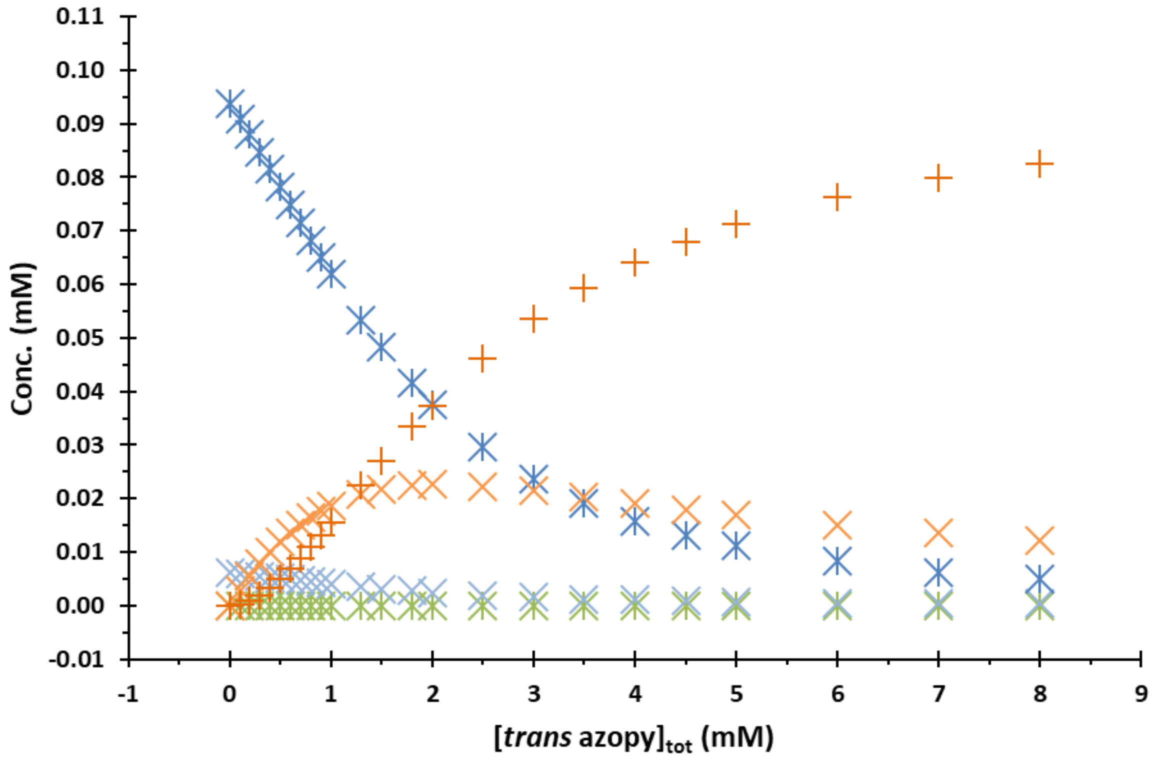

Supplementary Figure 32| Speciation plot for the titration of $FeTPPClO_4$ (0.1 Mm) in acetone containing DMSO-d_6 (25.87 mM) with *trans* azopyridine, using a model with the apparent binding constants of DMSO-d_6 and *trans* azopyridine (see Supplementary Table 11). Green: $FeTPP(acetone)_2^+$, light blue: $FeTPP(acetone)(DMSO)^+$, blue: $FeTPP(DMSO)_2^+$, light orange: $FeTPP(DMSO)(azopy)^+$, orange: $FeTPP(azopy)_2^+$.

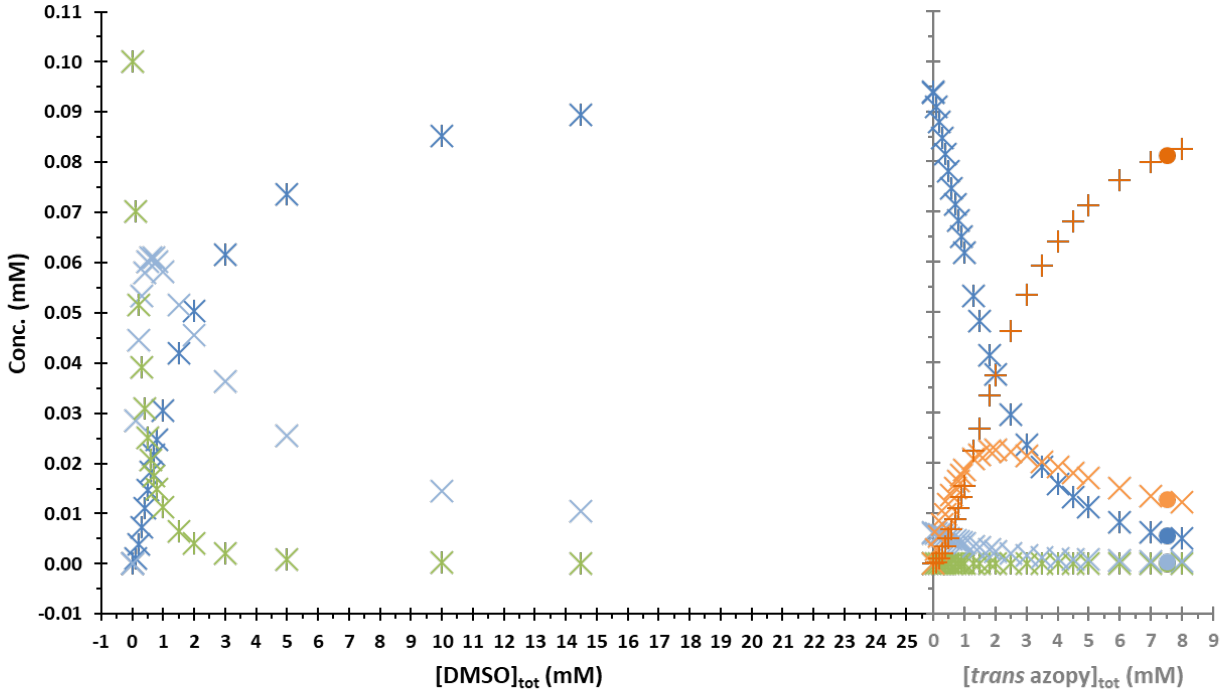

Supplementary Figure 33| Using the binding constants of DMSO-d_6 for the calculation of the binding constants of *trans* azopyridine, the concentrations of all relevant species in the titration experiments could be calculated. Left: Titration of 0.1 mM solution of $FeTPPClO_4$ in acetone with DMSO-d_6. Right: Titration of 0.1 mM solution of $FeTPPClO_4$ and 25.87 mM DMSO-d_6 in acetone with *trans* azopyridine. Dots: Composition of the solution containing 75 equivalents of *trans* azopyridine, used for the UV-Vis/far-Vis switching experiments (see 2.4). Green: $FeTPP(acetone)_2^+$, light blue: $FeTPP(acetone)(DMSO)^+$, blue: $FeTPP(DMSO)_2^+$, light orange: $FeTPP(DMSO)(azopy)^+$, orange: $FeTPP(azopy)_2^+$.

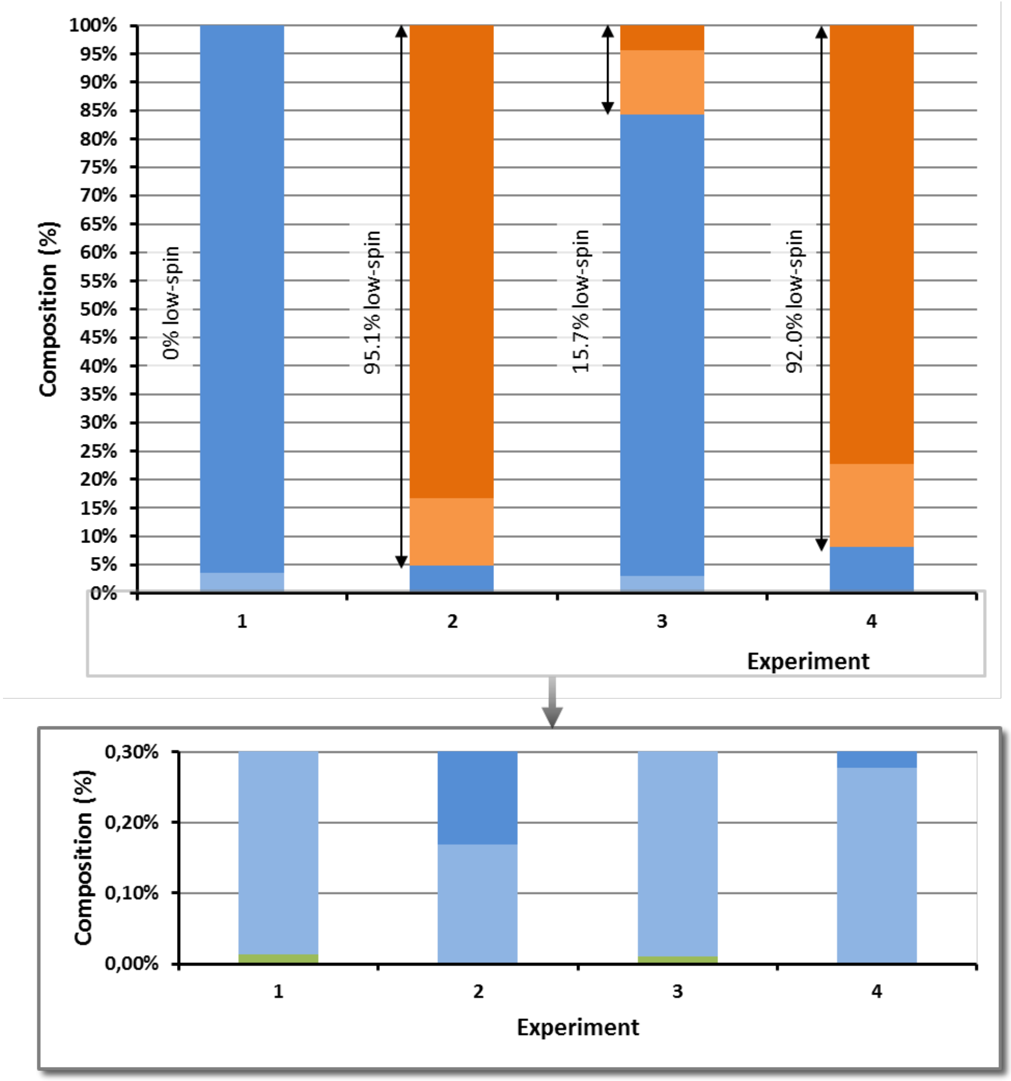

Supplementary Figure 34| Composition of the solutions of the NMR switching experiments as derived from Supplementary Table 12. Color code: blue: $FeTPP(DMSO)_2^+$, light blue: $FeTPP(acetone)(DMSO)^+$, orange: $FeTPP(azopy)_2^+$, light orange: $FeTPP(DMSO)(azopy)^+$. From the percentage of low-spin compounds after irradiation with 365 nm (Experiment 3, 15.7% low-spin) and 435 nm (Experiment 4, 92.0% low-spin) a switching efficiency of 76.3% results.

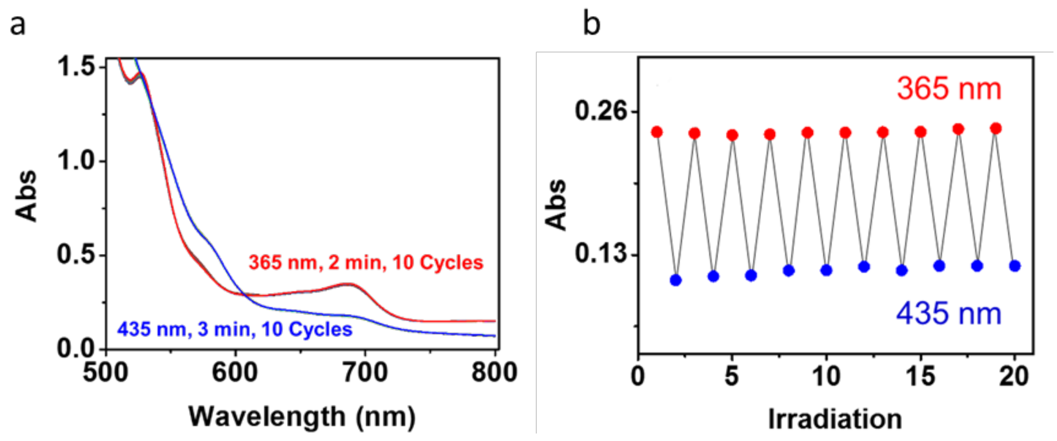

Supplementary Figure 35| Left: Visible spectra showing reversible change in absorption (up to 10 continuous irradiation cycles). To a 0.1 mM solution of $FeTPPClO_4$ in acetone, containing DMSO-d_6 (25.87 mM) *trans* azopyridine (75 eq., 7.5 mM) was added. Each switching cycle consisted of alternate irradiations using lights of wavelengths 365nm and 435 nm, respectively. Right: reversible changes in absorption at 686 nm as a function of the number of irradiation cycles.

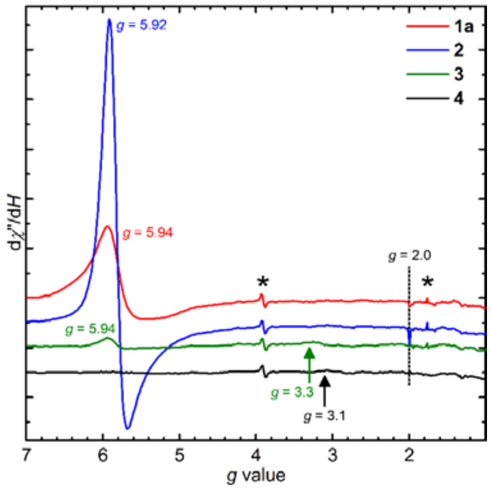

Supplementary Figure 36| X-band CW EPR spectra of 0.2 mM $FeTPPClO_4$ in acetone (**1a**), acetone and DMSO-d_6, (**2**), acetone, DMSO-d_6 and azopyridine (**3**), and acetone, DMSO-d_6, and 4-methoxypyridine (**4**). Asterisks denote the positions of residual resonator signals. Experimental parameters: microwave frequencies: 9.73 GHz; microwave power: 0.2 mW; modulation amplitude: 9.8 G; time constant: 82 ms; temperature: 8 K.

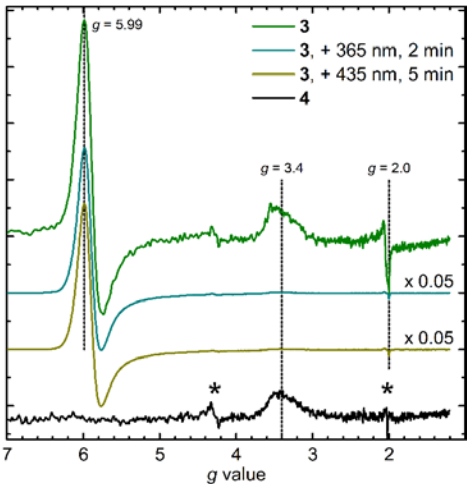

Supplementary Figure 37| X-band CW EPR spectra of 0.2 mM $FeTPPClO_4$ in CH_2Cl_2, DMSO-d_6 and azopyridine (**3**), and CH_2Cl_2, DMSO-d_6 and methoxypyridine (**4**). Spectra of complex **3** after illumination at 365 nm for 2 min and after further illumination at 435 nm for another 5 min are shown after 20-fold reduction in size. Asterisks denote the positions of residual resonator signals. Experimental parameters: microwave frequencies: 9.64 GHz; microwave power: 5 mW; modulation amplitude: 7.5 G; time constant: 164 ms; temperature: 4.8 K.

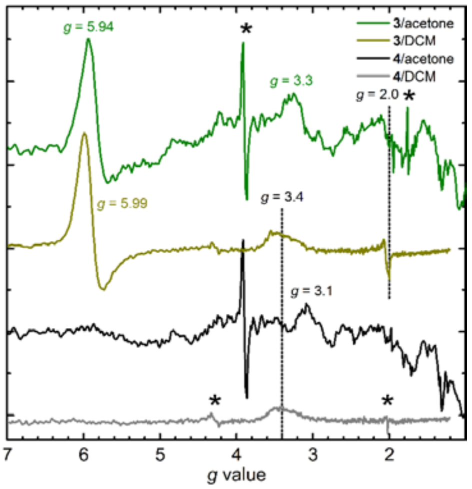

Supplementary Figure 38| Comparison of the X-band CW EPR spectra of **3** and **4** in samples with either acetone (see Fig. S36) or CH_2Cl_2 (see Fig. S37) as the solvent. Asterisks denote the positions of residual resonator signals. Experimental parameters: see Figs. S36, S37.

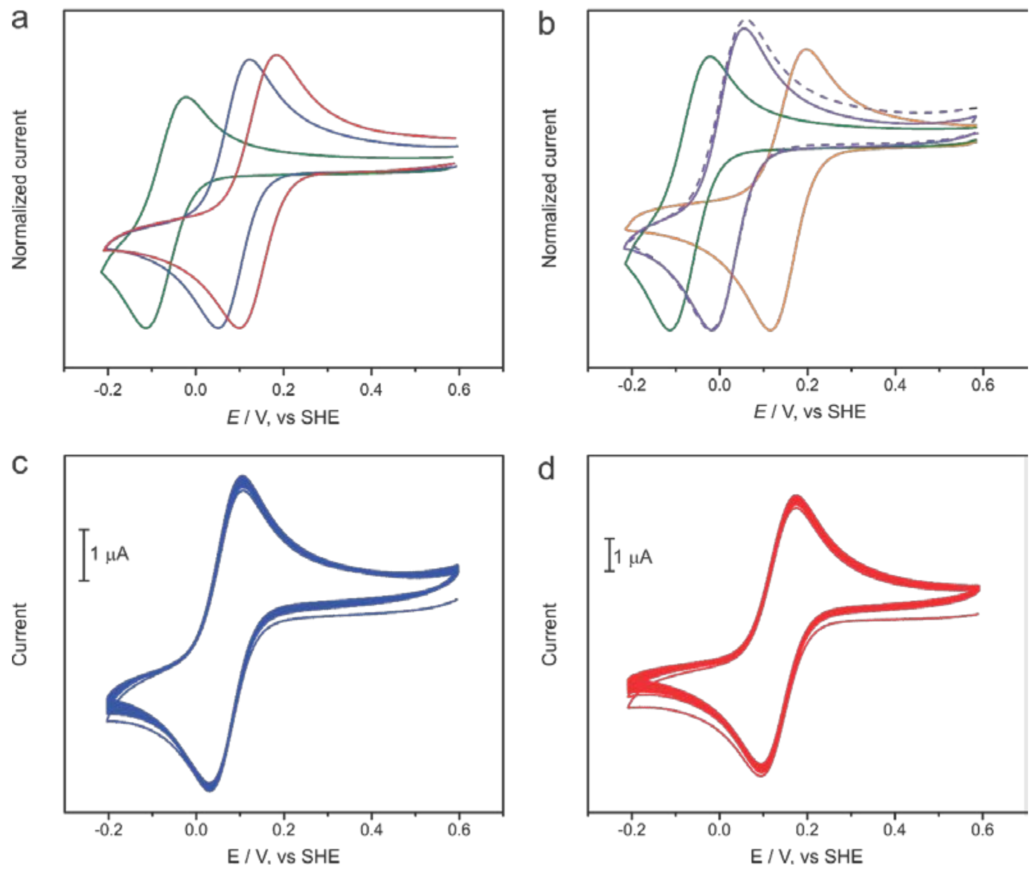

Supplementary Figure 39| Cyclic Voltammetry at 50 mV/s of Fe(III) porphyrins. (a) Comparison of the CVs obtained after the addition of 15 mM azopyridine to a 0.2 mM solution (5 mL) of $FeTPPClO_4$ in acetone containing DMSO (17.7 μL, blue), irradiating with a light of wavelength 365 nm for 20 min (red) and $FeTPPClO_4$ in acetone containing DMSO (17.7 μL, green). (b) Comparison of the CVs obtained for $FeTPPClO_4$ in acetone (orange), for $FeTPPClO_4$ in acetone containing DMSO (17.7 µL, green) and after the addition of excess 4-methoxypyridine (500 eq.) to a 0.2 mM solution (5 mL) of $FeTPPClO_4$ in acetone containing DMSO (17.7 μL, purple). The dashed purple line corresponds to the CV of the same solution after 20 min illumination with 365 nm light. (c) 50 consecutive CVs on the same solution as the blue CV in (a). (d) 50 consecutive CVs on the red solution from the (a) panel.

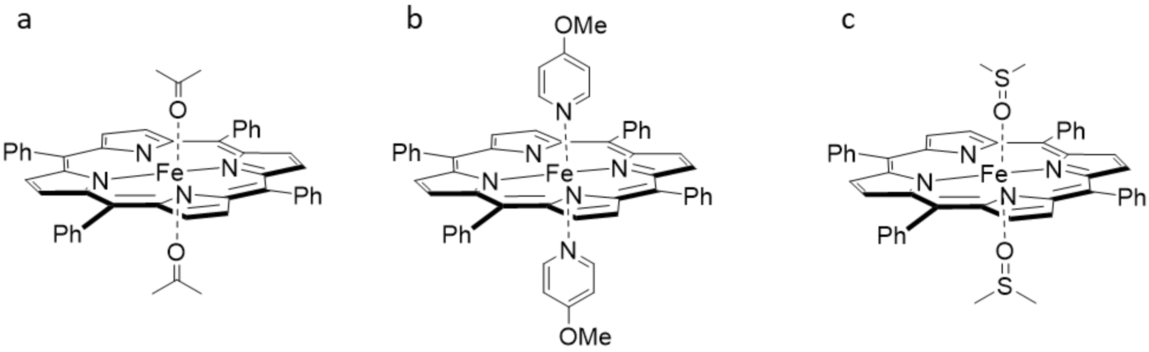

Supplementary Figure 40| Chemical structures of the ligand coordinated $FeTPP^+$.

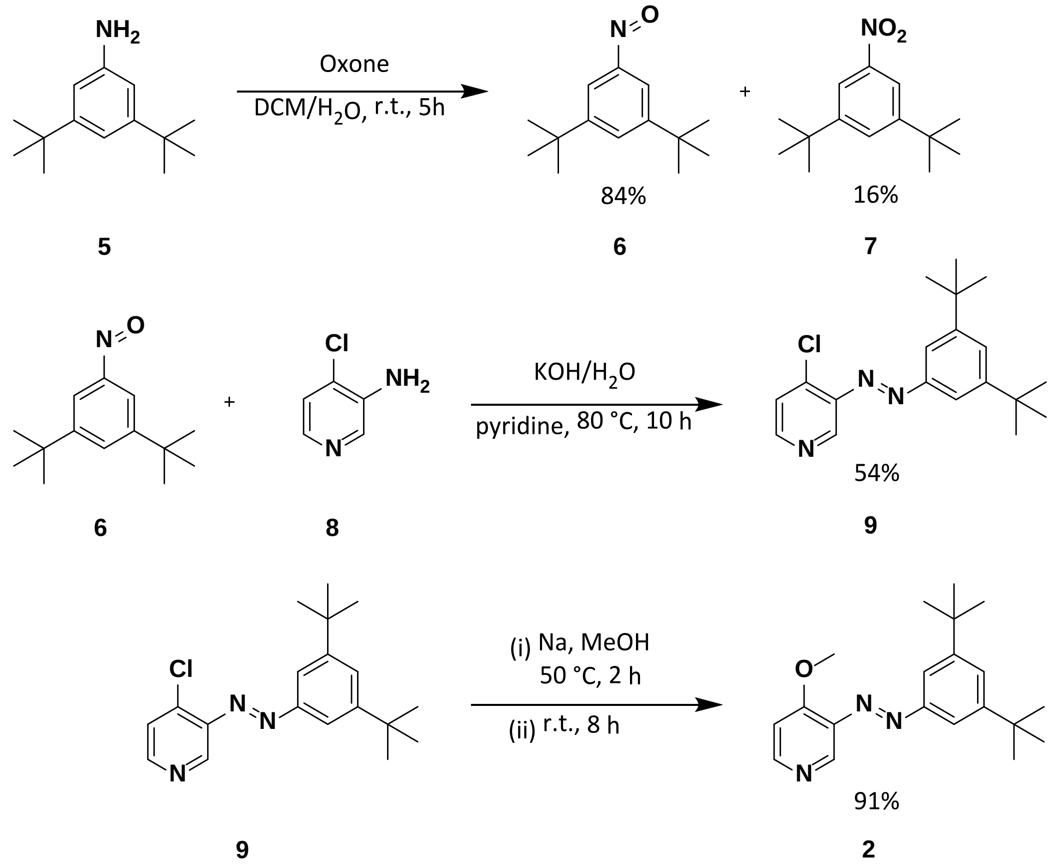

Supplementary Figure 41| Synthesis of the azopyridine **2**.

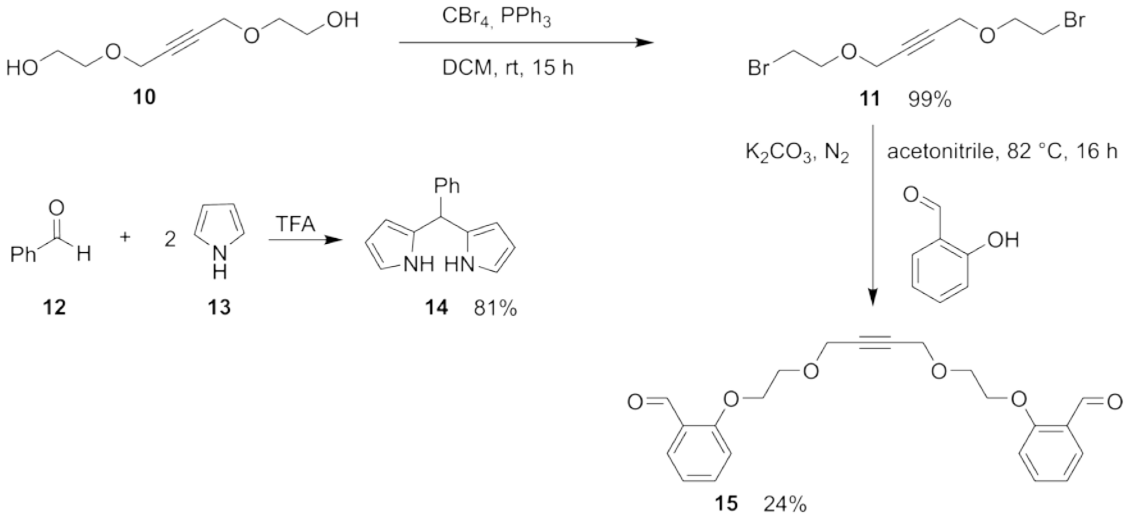

Supplementary Figure 42| Synthesis of the bridge **15** and the *meso*-phenyl dipyrromethane **14**.

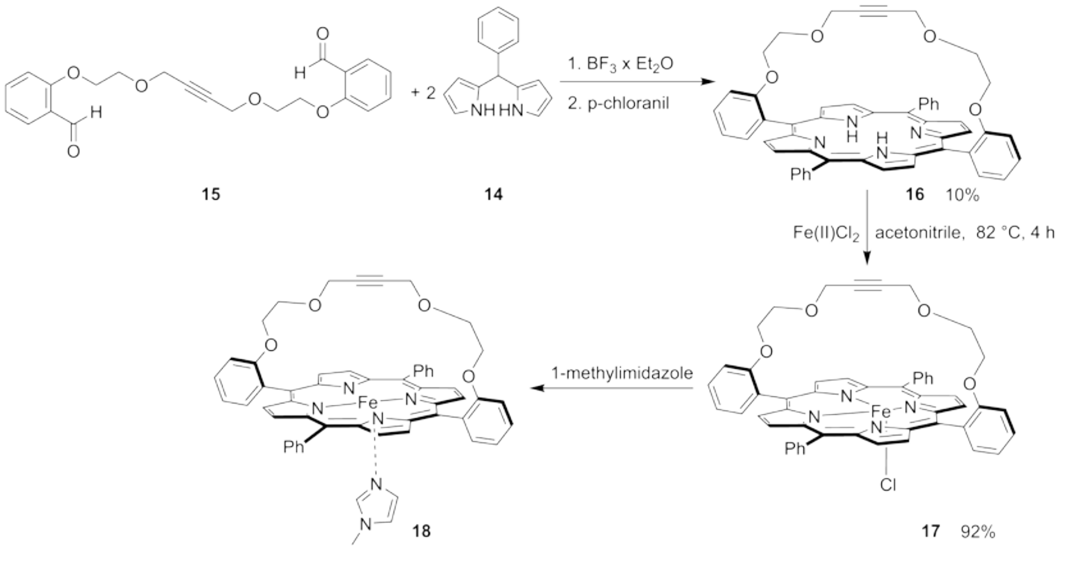

Supplementary Figure 43| Synthesis of the strapped iron porphyrin **17**.

3. Supplementary Tables

Supplementary Table 1| Photostationary equilibrium of azopyridine upon irradiation with lights of wavelengths 365 nm and 435 nm.

No.	Conditions	% *cis*	% *trans*
1	As synthesized, 60° C, 24 h		100
2	365 nm, 2 min	94.2	5.8
3	365 nm, 7 min	94.4	5.6
4	365 nm, 12 min	94.4	5.6
5	435 nm, 2 min	31.3	68.7
6	435 nm, 7 min	24.9	75.1
7	435 nm, 12 min	24.5	75.5

Supplementary Table 2| Relaxation Times (T_1 and T_2) of acetone and water at different concentrations of the high-spin complex $FeTPP(DMSO)_2^+$.

Concentration	Acetone		Water	
$FeTPP(DMSO)_2^+$ (mM)	T_1 (s)	T_2 (s)	T_1 (ms)	T_2 (ms)
0	5.95	5.34	3954	3240
0.2	5.22	4.44	135.4	113.3
0.5	3.73	3.44	86.1	71.9
1.0	2.62	2.46	57.6	47.0
1.5	2.12	1.91	46.9	36.6
2.0	1.68	1.51	36.9	29.9

Supplementary Table 3| Relaxation Times (T_1) of acetone and water at different concentrations of the low-spin complex $FeTPP(azopy)_2^+$.

Concentration	T_1	
$FeTPP(azopy)_2^+$ (mM)	Acetone (s)	Water (ms)
0	5.95	3954
0.2	5.9	1745
0.5	5.87	1329
1.0	5.62	*nd*
1.5	5.25	711.0
2.0	5.05	564.0

Supplementary Table 4| Observed and calculated phenyl and pyrrole shifts of the solution of 0.2 mM $FeTPPClO_4$ in CD_2Cl_2 upon addition of increasing amounts of acetone-d_6.

acetone$_{tot}$	δ_{Phenyl}						$\delta_{Pyrrole}$	
	p		*m*		*o*			
	obs.	calc.	obs.	calc.	obs.	calc.	obs.	calc.
(mM)	(ppm)		(ppm)		(ppm)		(ppm)	
0	8.07	8.07	11.67	11.67	9.91	9.91	-	-
100	8.20	8.20	11.46	11.50	10.17	10.19	-	-
200	8.34	8.34	11.30	11.34	10.47	10.47	-	-
400	8.61	8.62	11.04	11.05	11.04	11.02	-	-
800	9.04	9.05	10.66	10.65	11.84	11.88	-	-
1500	9.49	9.49	10.33	10.31	12.71	12.72	11.96	-
2000	9.68	9.65	10.22	10.21	13.07	13.03	16.79	-
4000	9.92	9.92	10.08	10.07	13.53	13.53	25.24	-
7500	10.03	10.03	10.03	10.04	13.73	13.73	30.02	-
11423	10.06	10.07	10.02	10.04	13.79	13.80	29.83	-
13600	10.14	-	10.05	-	13.91	-	40.16	-

Supplementary Table 5| Calculated composition of the solution of 0.2 mM $FeTPPClO_4$ in CD_2Cl_2 upon addition of increasing amounts of acetone-d_6.

acetone$_{tot}$	$FeTPPClO_4$	FeTPP(acetone)ClO_4	FeTPP(acetone)$_2^+$
(mM)	(mM)		
0	0.2	0	0
100	0.1825	0.0158	0.0017
200	0.1653	0.0286	0.0062
400	0.1338	0.0463	0.0199
800	0.0874	0.0605	0.0521
1500	0.0455	0.0591	0.0954
2000	0.0310	0.0536	0.1154
4000	0.0103	0.0357	0.1539
7500	0.0033	0.0217	0.1750
11423	0.0015	0.0149	0.1836
136000	0.0011	0.0127	0.1862

Supplementary Table 6| Calculated composition of the solution of 0.1 mM $FeTPPClO_4$ in acetone upon titration with DMSO-d_6 with the corresponding observed and calculated absorption.

$DMSO_{tot}$	Absorption		FeTPP(L1)(L2)$^+$			
			L1 / L2	acetone / acetone	acetone / DMSO	DMSO / DMSO
(mM)	obs.	calc.			(mM)	
0	0.1496	0.1496		0.1000	0	0
0.1	0.1703	0.1725		0.0703	0.0285	0.0012
0.2	0.1878	0.1880		0.0515	0.0446	0.0039
0.3	0.2007	0.1989		0.0393	0.0534	0.0074
0.4	0.2067	0.2070		0.0309	0.0580	0.0111
0.5	0.2150	0.2133		0.0250	0.0602	0.0147
0.6	0.2190	0.2183		0.0207	0.0610	0.0183
0.7	0.2220	0.2224		0.0174	0.0609	0.0216
0.8	0.2230	0.2259		0.0149	0.0603	0.0248
1.0	0.2325	0.2315		0.0113	0.0582	0.0306
1.5	0.2394	0.2409		0.0064	0.0516	0.0420
2.0	0.2472	0.2468		0.0042	0.0455	0.0503
3.0	0.2555	0.2539		0.0022	0.0363	0.0615
5.0	0.2600	0.2609		0.0009	0.0256	0.0735
10.0	0.2684	0.2672		0.0003	0.0146	0.0852
14.5	0.2684	0.2694		0.0001	0.0105	0.0894

Supplementary Table 7| Observed and calculated phenyl and pyrrole proton shifts upon titration of 0.2 mM $FeTPPClO_4$ in acetone-d_6 with increasing amounts of DMSO-d_6 utilising the binding constants derived from the UV-Vis titration experiments. Pyrrole shifts were calculated using 40.16 ppm for $FeTPP(acetone)_2^+$ (see Supplementary Table 4). Values marked in red could not be determined unambiguously because of severe line broadening (see Supplementary Figure 22b).

$DMSO_{tot}$	δ_{Phenyl}						$\delta_{Pyrrole}$	
	p		*m*		*o*			
	Obs.	Calc.	Obs.	Calc.	Obs.	Calc.	Obs.	Calc.
(mM)	(ppm)		(ppm)		(ppm)		(ppm)	
0	10.00	10.00	9.93	9.93	13.62	13.62	-	-
0.10	9.95	9.94	10.01	10.02	13.53	13.50	-	-
0.20	9.94	9.90	10.03	10.07	13.53	13.40	-	-
0.40	9.85	9.85	10.13	10.12	13.30	13.28	-	-
0.60	9.82	9.82	10.13	10.12	13.21	13.21	-	-
0.80	9.80	9.81	10.10	10.10	13.14	13.16	-	-
1.00	9.79	9.80	10.08	10.08	13.11	13.13	-	-
1.50	9.79	9.79	10.02	10.02	13.09	13.08	(59.35)	58.72
2.00	9.78	9.78	9.96	9.97	13.05	13.06	(60.88)	60.47
3.00	9.79	9.78	9.89	9.89	13.02	13.03	62.66	62.67
5.00	9.80	9.78	9.80	9.81	13.01	13.00	64.87	64.87
7.50	9.77	9.79	9.77	9.76	12.98	12.99	66.25	66.17
10.00	9.79	9.79	9.74	9.73	12.98	12.98	66.81	66.88
15.00	9.79	9.79	9.70	9.70	12.98	12.97	67.57	67.63
20.00	9.79	9.79	9.68	9.68	12.97	12.97	68.04	68.03
30.00	9.79	9.79	9.66	9.66	12.96	12.96	68.43	68.43
47.12	9.79	9.79	9.65	9.65	12.96	12.96	68.78	68.74

Supplementary Table 8| Calculated composition of the solution of 0.2 mM $FeTPPClO_4$ in acetone-d_6 upon titration with increasing amounts of DMSO-d_6, utilising the binding constants derived from the UV-Vis titration experiments.

$DMSO_{tot}$		FeTPP(L1)(L2)$^+$		
	L1<br>L2	acetone<br>acetone	acetone<br>DMSO	DMSO<br>DMSO
(mM)			(mM)	
0		0.20000	0	0
0.10		0.15269	0.04591	0.00140
0.20		0.11748	0.07734	0.00518
0.40		0.07319	0.11000	0.01682
0.60		0.04914	0.12070	0.03016
0.80		0.03511	0.12187	0.04302
1.00		0.02630	0.11897	0.05473
1.50		0.01472	0.10666	0.07862
2.00		0.00939	0.09429	0.09632
3.00		0.00477	0.07507	0.12016
5.00		0.00192	0.05245	0.14563
7.50		0.00090	0.03787	0.16122
10.00		0.00052	0.02958	0.16989
15.00		0.00024	0.02056	0.17921
20.00		0.00014	0.01574	0.18412
30.00		0.00006	0.01072	0.18922
47.12		0.00003	0.00693	0.19305

Supplementary Table 9| Change of the absorption (observed and calculated) of a solution of 0.1 mM $FeTPPClO_4$ in acetone-d_6 containing DMSO-d_6 (25.87 mM) upon titration with *trans* azopyridine. The composition of the solutions were calculated with a binding model using the apparent binding constants of both, DMSO-d_6 and *trans* azopyridine (see Supplementary Table 11).

trans azopyridine$_{tot}$	absorption		FeTPP(L1)(L2)$^+$					
			L1 / L2	acetone / acetone	acetone / DMSO	DMSO / DMSO	DMSO / azopy	azopy / azopy
(mM)	obs.	calc.				(mM)		
0	0.2939	0.2948		4.07E-05	6.13E-03	0.0938	0.0000	0.0000
0.1	0.2882	0.2882		3.95E-05	5.94E-03	0.0910	0.0028	0.0002
0.2	0.2808	0.2810		3.81E-05	5.74E-03	0.0879	0.0054	0.0009
0.3	0.2733	0.2735		3.67E-05	5.53E-03	0.0848	0.0077	0.0019
0.4	0.2671	0.2658		3.53E-05	5.32E-03	0.0815	0.0099	0.0033
0.5	0.2597	0.2580		3.38E-05	5.10E-03	0.0781	0.0118	0.0049
0.6	0.2534	0.2502		3.24E-05	4.88E-03	0.0748	0.0135	0.0068
0.7	0.2443	0.2425		3.09E-05	4.66E-03	0.0715	0.0151	0.0088
0.8	0.2335	0.2348		2.95E-05	4.45E-03	0.0682	0.0164	0.0109
0.9	0.2249	0.2274		2.81E-05	4.24E-03	0.0650	0.0176	0.0131
1.0	0.2120	0.2202		2.68E-05	4.04E-03	0.0619	0.0186	0.0154
1.3	0.2004	0.2001		2.30E-05	3.47E-03	0.0533	0.0208	0.0224
1.5	0.1901	0.1882		2.08E-05	3.14E-03	0.0482	0.0217	0.0269
1.8	0.1776	0.1724		1.79E-05	2.70E-03	0.0415	0.0224	0.0334
2.0	0.1634	0.1633		1.62E-05	2.45E-03	0.0376	0.0226	0.0374
2.5	0.1424	0.1446		1.27E-05	1.93E-03	0.0296	0.0223	0.0462
3.0	0.1335	0.1307		1.02E-05	1.54E-03	0.0236	0.0214	0.0534
3.5	0.1201	0.1202		8.23E-06	1.25E-03	0.0192	0.0203	0.0593
4.0	0.1110	0.1123		6.77E-06	1.02E-03	0.0158	0.0191	0.0641
4.5	0.1058	0.1062		5.64E-06	8.54E-04	0.0132	0.0180	0.0680
5.0	0.0996	0.1014		4.77E-06	7.22E-04	0.0111	0.0169	0.0712
6.0	0.0916	0.0946		3.51E-06	5.32E-04	0.0082	0.0150	0.0762
7.0	0.0910	0.0901		2.69E-06	4.07E-04	0.0063	0.0135	0.0799
8.0	0.0900	0.0870		2.12E-06	3.21E-04	0.0049	0.0122	0.0826

Supplementary Table 10| Stepwise binding constants for the formation of the complexes depicted in Supplementary Figure 23 and method of determination.

model	ligand	method	K_1''	K_2''	K_1'	K_2'	K_1	K_2
			(L mol^{-1})					
-	acetone-d_6	NMR	0.865	1.077	-	-	-	-
single[a)]	DMSO-d_6	UV-Vis	-	-	5862	596	-	-
combined[a,b)]	*trans* azopy	UV-Vis	-	-	5862	596	314	868

a) assuming that the acetone concentration is constant, because $FeTPP(acetone)_2^+$ is the predominating species in acetone solution and because acetone is in large excess. b) acetone concentration constant, variable concentration of DMSO.

Supplementary Table 11| Switching experiments in NMR solutions. Concentration of *trans* azopyridine in different experiments before (1) and after (2) the addition of *trans* azopyridine, and after irradiation with 365 nm (3) and 435 nm (4). The corresponding composition of the resulting solution is given in Supplementary Table 12.

Exp.	$FeTPPClO_{4,tot}$	$DMSO_{tot}$	*trans* $azopy_{tot}$	*cis* azopy	*trans* azopy	low-spin
	(mM)			(%)		
1	0.20	47.12	0	0	0	0
2	0.20	47.12	15.00	0	100	95.1
3	0.20	47.12	0.84	94.4	5.6	15.7
4	0.20	47.12	11.33	24.5	75.5	92.0

Supplementary Table 12| Switching experiments in NMR solutions. Calculated composition of the solutions in different experiments before (1) and after (2) the addition of *trans* azopyridine, and after irradiation with 365 nm (3) and 435 nm (4).

Exp.	$FeTPP(L1)(L2)^+$									
L1	acetone		acetone		DMSO		DMSO		azopy	
L2	acetone		DMSO		DMSO		azopy		azopy	
	(mM)	(%)	(mM)	(%)	(mM)	(%)	(mM)	(%)	(mM)	(%)
1	2.53E-05	0.01265	0.0069	3.464	0.1930	96.5	0	0	0	0
2	1.22E-06	0.00061	0.0003	0.168	0.0094	4.7	0.0238	11.9	0.1664	83.2
3	2.13E-05	0.01065	0.0058	2.919	0.1628	81.4	0.0226	11.3	0.0087	4.3
4	2.01E-06	0.00100	0.0006	0.277	0.0155	7.8	0.0295	14.7	0.1544	77.2

4. Supplementary References

1. Del Piero, S.; Melchior, A.; Polese, P.; Portanova, R. and Tolazzi, M. A Novel Multipurpose Excel Tool for Equilibrium Speciation Based on Newton-Raphson Method and on a Hybrid Genetic Algorithm. *Annali di Chimica* **96,** 29-49 (2006).
2. Bottemley, L. A. and Kadish, K. M. Counterion and Solvent Effects on the Electrode Reactions of Iron Porphyrins. *Inorg. Chem.* **20,** 1348-1357 (1981).
3. Nakamura, M.; Ohgo, Y. & Ikezaki, A. Electronic and magnetic structures of iron porphyrin complexes, Kadish, K. M. Smith & Guilard, R. (Eds.), Handbook of Porphyrin Science, vol. 7, World Scientific, Singapore (2010), pp. 1-146.
4. Walker, F. A.; Huynh, B. H.; Scheidt, W. R. & Osvath, S. R. *J. Am. Chem. Soc.* **108**, 5288-5297 (1986).
5. Watson, C. T.; Cai, S.; Shokhirev, N. V. & Walker, F. A. *Inorg. Chem.*, **44**, 7468–7484 (2005).
6. Boersma, A. D. ; Goff, H. M., Multinuclear Magnetic Resonance Spectroscopy of Spin-Admixed S=5/2, 3/2 Iron(III) Porphyrins. *Inorg. Chem.*, **21**, 581-586 (1982).
7. Adler, A. D. *et al.* A simplified synthesis for meso-tetraphenylporphine. *J. Org. Chem.* **32,** 476–476 (1967).
8. Adler, A. D.; Longo, F. R.; Kampas, F. and Kim, J. On the Preparation of Metalloporphyrins. *J. Inorg. Nucl. Chem.* **32,** 2443 (1970).
9. Reed, C. A. *et al.* The missing heme spin state and a model for cytochrome c'. The mixed S = 3/2, 5/2 intermediate spin ferric porphyrin: perchlorato(meso-tetraphenylporphinato)iron(III). *J. Am. Chem. Soc.* **101,** 2948–2958 (1979).
10. Thies, S.; Sell, H.; Bornhold, C.; Schütt, C.; Köhler, F.; Tuczek, F. and Herges, R., Light-Driven Coordination-Induced Spin-State Switching: Rational Design of Photodissociable Ligands. *Chem. Eur. J.* **18**, 16358-16368 (2012).
11. Littler, B. J. et al. Refined Synthesis of 5-Substituted Dipyrromethanes. *J. Org. Chem.* **64,** 1391-1396 (1999).
12. Cagnoni, A. J.; Varela, O.; Uhrig, M. L. and Kovensky, J. Efficient Synthesis of Thiolactoside Glycoclusters by Ruthenium-Catalyzed Cycloaddition Reaction of Disubstituted Alkynes on Carbohydrate Scaffolds. *Eur. J. Org. Chem.* **5,** 972-983 (2013).
